# Supplementary material for: Performance and resource requirements of in-person versus voice call versus automated telephone-based socioeconomic data collection modalities for community-based health programmes: a systematic review protocol
Source: BMJ Open. 2022 Apr 15;12(4):e057410. doi: 10.1136/bmjopen-2021-057410 (PMC9014069; doi:10.1136/bmjopen-2021-057410)
Supplement: Supplementary data [file bmjopen-2021-057410supp001.pdf]

Supplementary File

Search strategies

MEDLINE
Ovid MEDLINE(R) and Epub Ahead of Print, In-Process, In-Data-Review & Other Non-Indexed Citations and Daily

1. Telephone/
2. (telephone$ or phone$).tw.
3. ((voice or phone) adj1 call$).tw.
4. (phone adj2 interview$).tw.
5. Cell Phones/
6. Smartphone/
7. (phone$ adj1 (smart or cell)).tw.
8. (smartphone$ or cellphone$).tw.
9. (mobile adj2 (phone$ or device$)).tw.
10. or/1-9
11. Text Messaging/
12. (text or texts or texting).tw.
13. MMS.tw.
14. SMS.tw.
15. short message service.tw.
16. multimedia message service.tw.
17. (automated adj2 (telephone$ or text$ or message$ or questionnaire$)).tw.
18. (telephone adj1 administered adj1 questionnaire$).tw.
19. or/11-18
20. Interviews as Topic/
21. Patient Health Questionnaire/
22. Self Report/
23. (in adj1 person$).tw.
24. (in adj1 person$ adj4 (interview$ or survey$ or question$)).tw.
25. (face adj2 face adj4 (interview$ or survey$ or question$)).tw.
26. (face-to-face adj4 (interview$ or survey$ or question$)).tw.
27. or/20-26
28. 10 and 19
29. 10 and 27
30. 19 and 27
31. 10 and 19 and 27
32. 28 or 29 or 30 or 31
33. Vulnerable populations/ or socioeconomic factors/ or poverty/ or social class/ or Healthcare Disparities/ or Health Status Disparities/ or Poverty areas/ or Urban population/
34. (equit$ or inequit$ or inequalit$ or disparit$ or equality).tw.
35. (ethnic$ or race or racial$ or caste$).tw.

36. ((social$ or socio-economic or socioeconomic or economic or structural or material) adj3 (advantage$ or disadvantage$ or exclude$ or exclusion or include$ or inclusion or status or position or gradient$ or hierarch$ or class$ or determinant$)).tw.
37. (health adj3 (gap$ or gradient$ or hierarch$)).tw.
38. exp education/ or educational status/ or employment/ or income/ or occupations/ or social conditions/
39. (SES or SEP or sociodemographic$ or socio-demographic$ or demographic$ or income or wealth$ or poverty or affluen$).tw.
40. (educat$ adj3 (level$ or attain$ or status or well or better)).tw.
41. (occupation or unemploy$).tw.
42. (home owner$ or tenure).tw.
43. (household adj2 (income or wealth or status)).tw.
44. ((well or better or worse) adj2 off).tw.
45. or/33-43
46. Community Health Planning/
47. Community Health Services/
48. Community Health Nursing/
49. National Health Programs/
50. State Medicine/
51. Regional Health Planning/
52. Health Planning/
53. Health Plan Implementation/
54. Health Planning Guidelines/
55. Health Care Reform/
56. Health Resources/
57. Health Priorities/
58. Health Services Research/
59. "health services needs and demand"/
60. Needs Assessment/
61. State Health Plans/
62. Regional Health Planning/
63. Primary Health Care/
64. Health Services, Indigenous/
65. Rural Health Services/
66. Mobile Health Units/
67. randomized controlled trial/ or controlled clinical trials as topic/ or randomized controlled trials as topic/
68. (randomized or randomised or randomly or RCT).tw.
69. outcome assessment, health care/
70. comparative study/ or evaluation studies/ or meta-analysis/ or multicenter study/ or "systematic review"/ or validation studies/
71. epidemiologic studies/ or follow-up studies/ or longitudinal studies/ or prospective studies/ or controlled before-after studies/
72. or/46-71
73. 32 and 45 and 72

74. limit 73 to yr="1999 -Current"

Cochrane Library

#1 MeSH descriptor: [Telephone] this term only
#2 telephone* or phone*
#3 (voice or phone) near/1 call*
#4 phone near/2 interview*
#5 MeSH descriptor: [Cell Phone] this term only
#6 MeSH descriptor: [Smartphone] this term only
#7 phone* near/1 (smart or cell)
#8 smartphone* or cellphone*
#9 mobile near/2 (phone* or device*)
#10 #1 or #2 or #3 or #4 or #5 or #6 or #7 or #8 or #9
#11 MeSH descriptor: [Text Messaging] this term only
#12 text or texts or texting
#13 MMS or SMS
#14 "multimedia message service"
#15 "short message service"
#16 automated near/2 (telephone* or text* or message* or questionnaire*)
#17 telephone near/1 administered near/1 questionnaire*
#18 #11 or #12 or #13 or #14 or #15 or #16 or #17
#19 MeSH descriptor: [Interviews as Topic] this term only
#20 MeSH descriptor: [Patient Health Questionnaire] this term only
#21 MeSH descriptor: [Self Report] this term only
#22 in near/1 person*
#23 (in near/1 person* near/4 (interview* or survey* or question*))
#24 (face near/2 face near/4 (interview* or survey* or question*))
#25 (face-to-face near/4 (interview* or survey* or question*))
#26 #19 or #20 or #21 or #22 or #23 or #24 or #25
#27 #10 and #18
#28 #10 and #26
#29 #18 and #26
#30 #10 and #18 and #26
#31 #27 or #28 or #29 or #30
#32 MeSH descriptor: [Socioeconomic Factors] this term only
#33 MeSH descriptor: [Poverty] this term only
#34 MeSH descriptor: [Social Class] this term only
#35 MeSH descriptor: [Vulnerable Populations] this term only
#36 MeSH descriptor: [Healthcare Disparities] this term only
#37 MeSH descriptor: [Health Status Disparities] this term only
#38 MeSH descriptor: [Poverty Areas] this term only
#39 MeSH descriptor: [Urban Population] this term only
#40 equit* or inequit* or inequalit* or disparit* or equality

#41 ethnic* or race or racial* or caste*
#42 (social* or socio-economic or socioeconomic or economic or structural or material) near/3 (advantage* or disadvantage* or exclude* or exclusion or include* or inclusion or status or position or gradient* or hierarch* or class* or determinant*)
#43 health near/3 (gap* or gradient* or hierarch*)
#44 MeSH descriptor: [Education] explode all trees
#45 MeSH descriptor: [Educational Status] this term only
#46 MeSH descriptor: [Employment] this term only
#47 MeSH descriptor: [Income] this term only
#48 MeSH descriptor: [Occupations] this term only
#49 MeSH descriptor: [Social Conditions] this term only
#50 SES or SEP or sociodemographic* or socio-demographic* or income or wealth* or poverty or affluen*
#51 educat* near/3 (level* or attain* or status or well or better)
#52 occupation or unemploy*
#53 home owner* or tenure
#54 household near/2 (income or wealth or status)
#55 (well or better or worse) near/2 off
#56 #32 or #33 or #34 or #35 or #36 or #37 or #38 or #39 or #40 or #41 or #42 or #43 or #44 or #45 or #46 or #47 or #48 or #49 or #50 or #51 or #52 or #53 or #54 or #55
#57 MeSH descriptor: [Community Health Planning] this term only
#58 MeSH descriptor: [Community Health Services] this term only
#59 MeSH descriptor: [Community Health Nursing] explode all trees
#60 MeSH descriptor: [National Health Programs] this term only
#61 MeSH descriptor: [State Medicine] explode all trees
#62 MeSH descriptor: [Regional Health Planning] this term only
#63 MeSH descriptor: [Health Planning] this term only
#64 MeSH descriptor: [Health Plan Implementation] this term only
#65 MeSH descriptor: [Health Planning Guidelines] this term only
#66 MeSH descriptor: [Health Care Reform] this term only
#67 MeSH descriptor: [Health Resources] this term only
#68 MeSH descriptor: [Health Priorities] this term only
#69 MeSH descriptor: [Health Services Research] this term only
#70 MeSH descriptor: [Health Services Needs and Demand] this term only
#71 MeSH descriptor: [Needs Assessment] this term only
#72 MeSH descriptor: [State Health Plans] this term only
#73 MeSH descriptor: [Regional Health Planning] this term only
#74 MeSH descriptor: [Primary Health Care] this term only
#75 MeSH descriptor: [Health Services, Indigenous] this term only
#76 MeSH descriptor: [Rural Health Services] this term only
#77 MeSH descriptor: [Mobile Health Units] this term only
#78 #57 or #58 or #59 or #60 or #61 or #62 or #63 or #64 or #65 or #66 or #67 or #68 or #69 or #70 or #71 or #72 or #73 or #74 or #75 or #76 or #77
#79 #31 and #56 and #78 with Publication Year from 1999 to 2021, in Trials

Embase

1. telephone/
2. telephone interview/
3. (telephone$ or phone$).tw.
4. ((voice or phone) adj1 call$).tw.
5. (phone adj2 interview$).tw.
6. mobile phone/
7. smartphone/
8. (phone$ adj1 (smart or cell)).tw.
9. (smartphone$ or cellphone$).tw.
10. (mobile adj2 (phone$ or device$)).tw.
11. or/1-10
12. text messaging/
13. (text or texts or texting).tw.
14. MMS.tw.
15. SMS.tw.
16. multimedia message service.tw.
17. short message service.tw.
18. (automated adj2 (telephone$ or text$ or message$ or questionnaire$)).tw.
19. (telephone adj1 administered adj1 questionnaire$).tw.
20. or/12-19
21. interview/
22. (in adj1 person$).tw.
23. (in adj1 person$ adj4 (interview$ or survey$ or question$)).tw.
24. (face adj2 face adj4 (interview$ or survey$ or question$)).tw.
25. (face-to-face adj4 (interview$ or survey$ or question$)).tw.
26. or/21-25
27. 11 and 20
28. 11 and 26
29. 20 and 26
30. 11 and 20 and 26
31. 27 or 28 or 29 or 30
32. socioeconomics/
33. poverty/
34. social status/
35. social class/
36. vulnerable population/
37. health care disparity/
38. health disparity/
39. urban population/
40. (equit$ or inequit$ or inequalit$ or disparit$ or equality).tw.
41. (ethnic$ or race or racial$ or caste$).tw.

42. ((social$ or socio-economic or socioeconomic or economic or structural or material) adj3 (advantage$ or disadvantage$ or exclude$ or exclusion or include$ or inclusion or status or position or gradient$ or hierarch$ or class$ or determinant$)).tw.
43. (health adj3 (gap$ or gradient$ or hierarch$)).tw.
44. education/
45. educational status/
46. employment/
47. employment status/
48. unemployment/
49. household income/ or family income/ or income/
50. occupation/
51. (SES or SEP or sociodemographic$ or socio-demographic$ or demographic$ or income or wealth$ or poverty or affluen$).tw.
52. (educat$ adj3 (level$ or attain$ or status or well or better)).tw.
53. (occupation or unemploy$).tw.
54. (home owner$ or tenure).tw.
55. (household adj2 (income or wealth or status)).tw.
56. ((well or better or worse) adj2 off).tw.
57. or/32-56
58. public health/
59. health care planning/
60. community care/
61. community health nursing/
62. national health service/
63. health care policy/
64. health services research/
65. health service/
66. primary health care/
67. indigenous health care/
68. rural health care/
69. randomized controlled trial/ or controlled clinical trial/ or "randomized controlled trial (topic)"/
70. (randomized or randomised or randomly or RCT).tw.
71. outcome assessment/
72. comparative study/
73. evaluation study/
74. "systematic review"/ or "systematic review (topic)"/ or meta analysis/
75. epidemiology/
76. prospective study/
77. longitudinal study/
78. follow up/
79. or/58-78
80. 31 and 57 and 79
81. limit 80 to yr="1999 -Current"

Global Health

1. mobile telephones/ or telephones/
2. (telephone$ or phone$).tw.
3. ((voice or phone) adj1 call$).tw.
4. (phone adj2 interview$).tw.
5. (phone$ adj1 (smart or cell)).tw.
6. (smartphone$ or cellphone$).tw.
7. (mobile adj2 (phone$ or device$)).tw.
8. or/1-7
9. (text or texts or texting).tw.
10. (MMS or SMS).tw.
11. multimedia message service.tw.
12. short message service.tw.
13. (automated adj2 (telephone$ or text$ or message$ or questionnaire$)).tw.
14. (telephone adj1 administered adj1 questionnaire$).tw.
15. or/9-14
16. interviews/
17. (in adj1 person).tw.
18. (in adj1 person adj4 (interview$ or survey$ or question$)).tw.
19. (face adj2 face adj4 (interview$ or survey$ or question$)).tw.
20. (face-to-face adj4 (interview$ or survey$ or question$)).tw.
21. or/16-20
22. 7 and 15
23. 7 and 21
24. 15 and 21
25. 7 and 15 and 21
26. 22 or 23 or 24 or 25
27. socioeconomic status/ or socioeconomics/
28. poverty/
29. exp social classes/ or caste/ or social inequalities/ or social mobility/
30. urban population/
31. (equit$ or inequit$ or inequalit$ or disparit$ or equality).tw.
32. (ethnic$ or race or racial$ or caste$).tw.
33. ((social$ or socio-economic or socioeconomic or economic or structural or material) adj3 (advantage$ or disadvantage$ or exclude$ or exclusion or include$ or inclusion or status or position or gradient$ or hierarch$ or class$ or determinant$)).tw.
34. (health adj3 (gap$ or gradient$ or hierarch$)).tw.
35. education/
36. employment/
37. occupations/
38. income/ or household income/
39. living conditions/
40. (SES or SEP or sociodemographic$ or socio-demographic$ or demographic$ or income or wealth$ or poverty or affluen$).tw.

41. (educat$ adj3 (level$ or attain$ or status or well or better)).tw.
42. (occupation or unemploy$).tw.
43. (home owner$ or tenure).tw.
44. (household adj2 (income or wealth or status)).tw.
45. ((well or better or worse) adj2 off).tw.
46. or/27-45
47. 26 and 46
48. limit 47 to yr="1999 -Current"

ClinicalTrials.gov

Search 1
socioeconomic AND (telephone OR phone) AND (interview OR face-to-face OR in-person) AND community | Interventional Studies

Search 2
socioeconomic AND (telephone OR phone) AND (text OR SMS OR MMS) AND community | Interventional Studies

Search 3
socioeconomic AND (text OR SMS OR MMS) AND (interview OR face-to-face OR in-person) AND community | Interventional Studies

WHO ICTRP

Search 1
socioeconomic AND telephone AND interview AND community

Search 2
socioeconomic AND telephone AND text AND community

Search 3
socioeconomic AND text AND interview AND community

OpenGrey

socioeconomic AND (telephone OR phone OR text OR interview OR face-to-face OR in-person) AND community
