# Supplementary material for: Performance and resource requirements of in-person versus voice call versus automated telephone-based socioeconomic data collection modalities for community-based health programmes: a systematic review protocol
Source: BMJ Open. 2022 Apr 15;12(4):e057410. doi: 10.1136/bmjopen-2021-057410 (PMC9014069; doi:10.1136/bmjopen-2021-057410)
Supplement: Author's manuscript [file bmjopen-2021-057410.draft_revisions.pdf]

BMJ Open is committed to open peer review. As part of this commitment we make the peer review history of every article we publish publicly available.

When an article is published we post the peer reviewers' comments and the authors' responses online. We also post the versions of the paper that were used during peer review. These are the versions that the peer review comments apply to.

The versions of the paper that follow are the versions that were submitted during the peer review process. They are not the versions of record or the final published versions. They should not be cited or distributed as the published version of this manuscript.

BMJ Open is an open access journal and the full, final, typeset and author-corrected version of record of the manuscript is available on our site with no access controls, subscription charges or pay-per-view fees (http://bmjopen.bmj.com).

If you have any questions on BMJ Open's open peer review process please email info.bmjopen@bmj.com

BMJ Open

Performance and resource requirements of in-person, voice call, and automated telephone-based socioeconomic data collection modalities for community-based health programmes: a systematic review protocol

Journal:	*BMJ Open*
Manuscript ID	bmjopen-2021-057410
Article Type:	Protocol
Date Submitted by the Author:	14-Sep-2021

Complete List of Authors:	Allen, Luke; London School of Hygiene & Tropical Medicine, Department of Clinical Research<br>Mackinnon, Shona; University of Glasgow<br>Gordon, Iris; London School of Hygiene & Tropical Medicine, Department of Clinical Research<br>Blane, David; University of Glasgow<br>Marques, Ana Patricia; London School of Hygiene and Tropical Medicine International Centre for Eye Health,<br>Gichuhi, Stephen; University of Nairobi<br>Mwangi, Alice<br>Burton, Matthew J; London School of Hygiene & Tropical Medicine, Department of Clinical Research<br>Bolster, Nigel; Peek Vision<br>Macleod, David; London School of Hygiene & Tropical Medicine<br>Kim, Min; London School of Hygiene and Tropical Medicine Faculty of Infectious and Tropical Diseases<br>Ramke, Jacqueline; London School of Hygiene & Tropical Medicine, Department of Clinical Research<br>Bastawrous, Andrew

Keywords:	PRIMARY CARE, PUBLIC HEALTH, Epidemiology < TROPICAL MEDICINE

SCHOLARONE™
Manuscripts

Performance and resource requirements of in-person, voice call, and automated telephone-based socioeconomic data collection modalities for community-based health programmes: a systematic review protocol

Authors

Dr Luke N Allen (corresponding author)
International Centre for Eye Health, Department of Clinical Research, London School of Hygiene & Tropical Medicine, Keppel St, London WC1E 7HT
luke.allen@lshtm.ac.uk

Dr Shona Mackinnon
NHS Education for Scotland
shonamackinnon@doctors.org.uk

Ms Iris Gordon
International Centre for Eye Health, Department of Clinical Research, London School of Hygiene & Tropical Medicine, Keppel St, London WC1E 7HT
ORCID: 0001-8143-8132
iris.gordon@lshtm.ac.uk

Dr David Blane
Institute of Health and Wellbeing, University of Glasgow
David.Blane@glasgow.ac.uk
ORCID ID: 0000-0002-3872-3621

Dr Ana Patricia Marques
International Centre for Eye Health, Department of Clinical Research, London School of Hygiene & Tropical Medicine, Keppel St, London WC1E 7HT
Patricia.Marques@lshtm.ac.uk
ORCID ID: 0000-0001-8242-7021

Dr Stephen Gichuhi
MBChB, M.Med (Ophth), MBA, MSc (Epid), PhD, FCOphth(ECSA)
Senior Lecturer & Consultant Ophthalmologist, Chairman, Department of Ophthalmology, University of Nairobi
sgichuhi@uonbi.ac.ke

Ms Alice Mwangi
Operation Eyesight
mwangia@operationeyesight.com

Prof Matthew J. Burton

For peer review only

International Centre for Eye Health, Department of Clinical Research, London School of Hygiene & Tropical Medicine, Keppel St, London WC1E 7HT
matthew.burton@lshtm.ac.uk

Dr Nigel Bolster
Peek Vision, 90a High Street, Berkhamsted, Hertfordshire, England HP4 2BL
International Centre for Eye Health, Department of Clinical Research, London School of Hygiene & Tropical Medicine, Keppel St, London WC1E 7HT
ORCID: 0000-0001-6607-1723

nigel@peekvision.org

Dr David Macleod
International Statistics & Epidemiology Group, Department of Infectious Disease Epidemiology, London School of Hygiene & Tropical Medicine, Keppel St, London WC1E 7HT
david.macleod@lshtm.ac.uk

Ms Min Kim
International Centre for Eye Health, Department of Clinical Research, London School of Hygiene & Tropical Medicine, Keppel St, London WC1E 7HT
min.kim@lshtm.ac.uk

Assoc Prof Jacqueline Ramke
International Centre for Eye Health, Department of Clinical Research, London School of Hygiene & Tropical Medicine, Keppel St, London WC1E 7HT
School of Optometry and Vision Science, University of Auckland, Auckland, New Zealand
Jacqueline.Ramke@lshtm.ac.uk

Assoc Prof Andrew Bastawrous
International Centre for Eye Health, Department of Clinical Research, London School of Hygiene & Tropical Medicine, Keppel St, London WC1E 7HT
Andrew.bastawrous@lshtm.ac.uk

For peer review only

Abstract

Introduction

Gathering data on socioeconomic status (SES) is a prerequisite for any health programme that aims to assess and improve the equitable distribution of its outcomes. Many different modalities can be used to collect SES data, ranging from (1) face-to-face elicitation, to (2) telephone-administered questionnaires, to (3) automated text message-based systems. The relative costs and perceived benefits to patients and providers of these different data collection approaches is unknown. This review aims to compare the resource requirements, performance characteristics, and acceptability to participants and service providers of these three approaches to collect SES data from those enrolled in health programmes.

Methods and analysis

An information specialist will conduct searches on the Cochrane Library, MEDLINE, Embase, Global Health, Clinicaltrials.gov, the WHO ICTRP and OpenGrey. All databases will be searched from 1999 to present with no language limits used. We will also search Google Scholar and check the reference lists of relevant articles for further potentially eligible studies. Any empirical study design will be eligible. Two reviewers will independently screen titles, abstracts and full-text articles; and complete data extraction. For each study we will extract data on the modality characteristics, primary outcomes (response rate, completeness, and equivalence, time and cost requirements) and secondary outcomes (acceptability to patients and providers). We will synthesise findings thematically without meta-analysis.

Ethics and dissemination

Ethical approval is not required, as our review will include published and publicly accessible data. This review is part of a project to improve equitable access to eye care services in low- and middle-income countries. However, the findings will be useful to policymakers and programme managers in a range of health- and non-health settings. We will publish our findings in a peer-reviewed journal and develop an accessible summary of results for website posting and stakeholder meetings.

Registration

This review is registered with PROSPERO CRD42021251959

Strengths and limitations of this study

- As far as we are aware, this review will be the first to directly compare three commonly used data collection modalities for the collection of SES data.
- The review will be comprehensive, covering published and grey literature in any language.
- This review will be robust, using independent dual review at every stage, and following best-practice guidelines.
- There may only be a small number of articles in the literature that compare the different modalities head-to-head and provide data on the outcomes of interest.

Introduction

Rationale

For peer review only

Inequalities in health are pervasive and stubbornly persistent. Individuals with lower levels of income, education, and social status tend to experience the worst health outcomes irrespective of where they are in the world.[1] In 1971 Julian Tudor Hart observed that the availability of good medical care tends to vary inversely with the need for it in the population served.[2] This inverse care law manifests in the majority of global health and development programmes where individuals with the lowest socioeconomic status (SES) tend to face the highest barriers in accessing care and are the least likely to attain good outcomes.

Recognising marked inter- and intra-national disparities in health outcomes, the World Health Organisation (WHO) was constituted in 1948 with the mandate of advancing 'health for all'.[3] The contemporary manifestation of this mission is encapsulated in the concept of Universal Health Coverage (and Sustainable Development Goal target 3.8[4]) which seeks to extend coverage to disenfranchised groups. Emerging emphases on attaining *effective* coverage,[5] and *equitable* coverage[6 7] seek to shift the success criteria from supply-side provision of services to demand-side receipt of effective services according to need. These trends are underpinned by the principle of 'proportionate universalism': seeking to improve the health of all, with the greatest gains experienced by those with the greatest needs.[8] There is also an increasing interest in understanding the distribution of programme benefits across socio-demographic groups - for instance women, those living in rural locations, and those living in conditions of poverty.[9]

All attempts to boost equity in service provision are predicated on adequate collection and analysis of sociodemographic data. Previous work has demonstrated that sociodemographic data can be collected using a variety of modalities in the community setting including in-person, telephone voice calls, and using automated telephone-based systems[10] (Box 1). However, as far as we are aware, the relative costs and benefits of the different modalities have not been studied, including the skills, equipment, time and financial resources required and acceptability to data collectors and service beneficiaries.

This review aims to answer the research question 'how do three common SES data collection modalities compare in terms of performance characteristics, resource requirements and acceptability to participants and service providers?' Selecting an appropriate and cost-effective modality is an important first step towards advancing equitable effective service coverage.

The findings of this review will directly inform the development of school and community-based eye health screening programmes that operates in several low- and middle-income countries (LMICs) including Botswana, Ethiopia, Kenya, Nepal, Pakistan, Tanzania, Uganda and Zimbabwe.[11] However, the collection of SES data is relevant for a much wider range of global health programmes, as well as non-health programmes aimed at improving educational, agricultural, gender equity, and economic outcomes, among others.

Descriptions of the interventions
Three different modalities for SES data collection constitute the interventions of interest for this review: in-person; voice call; and automated telephone data collection. Box 1 provides the definition for each.

For peer review only

Box 1: Definitions of the three data collection approaches used in this review

In-person data collection includes any form of exchange between a programme implementer and a participant or their responsible guardian, whereby the programme implementer asks predefined questions to ascertain the participants' socioeconomic status and a synchronous response is received i.e., both parties occupy the same time and space, and the response is recorded by the implementer before the encounter is terminated. Any recording modality used by the programme implementer will be included, such as pen and paper or completion of an electronic form. For this review we will also include self-administered questionnaires as a subtype of in-person data collection, provided that; the data collection instrument is provided when the participant presents to a programme implementer in-person; the participant is asked to complete the data entry form; and the participant submits their responses before departing.

Voice call data collection includes real-time, telephone-based verbal exchanges between programme implementers and participants whereby SES data is elicited and recorded by the programme implementer using predefined questions. Videocalls will be included as a subtype of voice-calls.

Automated telephone-based data collection includes any mobile-telephone-based asynchronous exchange of information whereby participants are sent a standardised text message (SMS), multimedia message (MMS), email, or automated phone call and asked to provide SES data. Responses can be provided using the same modality or any other digital form e.g. entering details on a webpage. Interventions that require participants to engage with human programme implementers will be excluded. All forms of phrasing of the requests and responses will be included.

Other terminology used in this review

'Community-based health programmes'. For the purpose of this review, health programmes are defined as organised activities to improve one or more health outcome(s) in a defined population. Community-based care encompasses all settings except hospitals. Other definitions of community-based care exclude primary care facilities,[12] but these will be included in this review, along with outreach/mobile clinics, community centres, schools, workplaces, and people's own homes.

'Programme implementers'. Anyone with a formal responsibility to collect data on behalf of the health programme will be dubbed a 'programme implementer' for the purpose of this review. This term will cover voluntary and paid staff, and all cadre types.

'Participants'. Any health programme beneficiary/recipient/client/patient that is asked to provide their SES data will be dubbed a 'participant' for the purpose of this review.

'Socioeconomic status' (SES). Socioeconomic status is a critically important but nebulous concept that pertains to social and economic standing within society.[13] It determines exposure to the social determinants of health; "the conditions in which people are born, grow, live, work, and age",[14] and relates to issues of privilege, power and control.[15] Almost all health outcomes are patterned

For peer review only

according to SES, with the most disadvantaged populations experiencing the worst health outcomes.[16 17 18] SES is commonly measured using income, education, occupation, and other metrics such as wealth, caste, and place of residence. We will include all of these domains, as well as any other proxies that are identified by researchers as capturing SES.

'Low- and middle-income countries' (LMICs): Just as health inequalities exist within countries - driven by differential access to resources, power, privilege and control - the same set of factors drive international health inequalities. In 1975 Samuel Preston found that national life expectancy was tightly correlated with gross domestic product (GDP) by purchasing power parity, following a logarithmic path whereby small rises in GDP are initially associated with large gains in life expectancy, followed by increasingly diminishing returns.[19 20] In 1978 the World Bank first divided countries into 'low' and 'middle-income' groupings, based on gross national income (GNI) per capita. Whereas GDP captures the total value produced in a nation, GNI also includes net income received from overseas. Despite the fact that national finances are a fairly crude proxy,[21 22] many development agencies have come to use the World Bank categorisations to define eligibility for support. This review will use the World Bank analytic classifications for fiscal year 2021; defining LMICs as countries with gross national income (GNI) per capita ≤ $12,535[23] using the Atlas method.[24]

Objectives
We aim to systematically review the findings of empirical studies that have compared in-person vs voice call vs telephone-based modalities for gathering SES data for community-based health programmes in terms of their resource requirements, performance characteristics, and acceptability to participants and service providers. We will include studies that compare any of these modalities. Our findings should help programme managers make evidence-informed decisions when selecting the most appropriate modality for SES data collection.

Methods and analyses

This protocol is reported according to the relevant sections of the Preferred Reporting Items for Systematic Review and Meta-Analysis Protocols (PRISMA-P) guidelines.[25] It has been registered with PROSPERO, CRD42021251959.

Population
For this methodological paper, the 'population' is composed of studies rather than people, namely those that seek to compare two or more modalities for socioeconomic data collection from individuals enrolled in health programmes. Studies that only report on one mode of data collection will be excluded.

Interventions
The interventions being studied are three different modalities for collecting socioeconomic data. The focus is on the modality of data collection (e.g. in-person vs voice call vs automated) rather than the content of the wording that is used to elicit information.

Three different modalities for SES data collection constitute the interventions of interests for this review: in-person, voice-call, and automated telephone systems, as defined in Box 1.

For peer review only

Studies that gather SES data at the household or community level will only be included if these data are used to make assumptions about the SES of identifiable individual participants enrolled (or due to be enrolled) in the service delivery programme of interest. Any number of modalities can be studied. There is no index/gold-standard data collection modality. Interventions that bundle requests for SES data with requests for other data (e.g., broader demographic data) will be included, as long as separate results are reported for the SES data collection element. Interventions that use a blend of two or more modalities to request or receive data will be excluded. Whilst automated email-based SES elicitation has not been built into the search strategy we will include studies that use this approach.

Comparator

In-person, voice call, and automated telephone system attributes will be compared against each other.

Primary outcomes

There are two groups of primary outcomes; performance characteristics and resource requirements.

Performance characteristics

- **Response rate**: number of completed forms divided by the total number of elicitation attempts.

- **Completeness**: proportion of missing items.

- **Equivalence:** [For studies that compare two or more approaches] agreement between the responses obtained from two or more different modalities. This will be calculated at the overall level of the SES questionnaire, rather than at the individual item level. Following Belisario and Gwaltney, we will use comparisons of mean scores between modalities and/or correlations and/or measures of agreement - which include intra-class correlation (ICC) coefficients, Pearson product-moment correlations, Spearman rho and weighted Kappa coefficients.

Resource requirements

- **Time:** the time taken to gather SES data using each approach (range and mean).
- **Costs:** any financial data on the costs of operating the data collection approach will be included. Fixed costs include the costs of equipment, software, insurance, and personnel required to set up a given data elicitation modality. We will also include any ongoing support costs. We will aim to calculate the fixed and per-person costs to purchasers.

Secondary outcome

Acceptability to participants and service providers: Survey or interview results reporting on how programme implementers and participants feel about the data collection modality in terms of intrusiveness, ease of use, time requirement, and general acceptability, as well as perceived advantages, barriers, disadvantages, and additional costs presented by the beneficiaries, data

For peer review only

collectors, or study authors. This includes an assessment of socioeconomic barriers to accessing the modalities.

Study types to be included

All empirical study designs that present outcome data on the data collection modalities will be included. Studies that use simulated data, or data obtained from populations other than the intended beneficiaries will be excluded. Both quantitative and qualitative study designs will be included as long as they report on one or more of the outcomes of interest. Although the search strategy has been designed to capture comparative methodological studies, articles that present the outcomes for single approaches will also be included for a secondary analysis. Review articles will not be included, but their primary studies will be screened for potential inclusion.

Search methods for identification of studies

Search strategy

The search strategy will be built around three blocks: the three data collection modalities, SES concepts, and study design or study setting terms. The search will be limited to human studies published since 1999: the year that it first became possible to send cross-network SMS messages. We will search for full-text studies published in any language. We will not include reports of studies published as conference abstracts. The full search strategies used for each database are presented in the Appendix.

Electronic databases

We will search the following information resources: the Cochrane Library, MEDLINE, Embase and Global Health. We will search ClinicalTrials.gov and the WHO International Clinical Trials Registry Platform (ICTRP) for current and ongoing trials. OpenGrey will be searched for grey literature. The first 20 pages of Google Scholar will also be screened. We will check the reference lists of included studies and relevant systematic reviews to identify any additional potentially relevant reports of studies. Key authors will be contacted to uncover additional or upcoming studies.

Measures of effect

We will calculate mean differences for methodological performance between the modalities, as well as for time and cost differences. For equivalence we will follow Belisario[26] and Gwaltney,[27] using comparisons of mean scores between modalities and/or correlations and/or measures of agreement - which include intra-class correlation (ICC) coefficients, Pearson product-moment correlations, Spearman rho and weighted Kappa coefficients.

Data collection and analysis

Selection of studies

Initial screening of studies will be based on the information contained in their titles and abstracts, using online software (Covidence systematic review software, Veritas Health Innovation, Melbourne, Australia. Available at www.covidence.org). Studies that clearly do not meet the inclusion criteria will be excluded. The first 10% of papers will be screened by two reviewers collaboratively to align interpretation of the inclusion criteria and clarify the wording as appropriate. Any changes or

For peer review only

amendments will be recorded. All remaining records will be screened independently by two reviewers. They will meet after every 10% batch of papers has been screened to discuss any issues. Any disagreements will be resolved through consensus-based discussion, or if necessary, discussion with a third reviewer.

We will obtain full texts for the potentially relevant papers. Two review authors will independently assess the papers against the inclusion criteria to determine their eligibility for inclusion. Non-English language papers will be translated into English. The review authors will resolve disagreements through consensus-based discussion, or if necessary, discussion with a third reviewer. The reviewers will record reasons for exclusion at the full-text screening stage. A PRISMA flow diagram will be completed to summarise the study selection process.[28]

Data extraction and management

Two review authors will independently extract study characteristics and data from the included studies using a custom Excel data extraction form based on the Cochrane template for RCTs and non RCTs.[29] The data extraction form will be piloted on 30 studies by two review authors and required amendments will be made by consensus. We anticipate a broad scope of included studies, so data charting will be an iterative process throughout the review, with agreement calculated and discussed at regular intervals (after each 10% batch of studies) and the data extraction form will be amended as required. Any discrepancies will be resolved by discussion, and a third reviewer will be consulted if necessary.

The following data will be extracted:

- Article title
- Journal title
- Authors
- Country
- Language
- Publication year
- Type of study
- Focus of the service delivery programme
- Sociodemographic characteristics for the population served: age, sex, urban/rural, ethnicity, marital status
- Number of participants
- Questions used to assess SES
- Point at which SES data are being collected: start, mid-point, or end of programme
- Number of times SES data are collected from each participant
- Types of intervention, including:
    - modality
    - who gathers the SES data
    - when in the patient journey
    - equipment used
    - who provides the data
    - whether data collection is synchronous or asynchronous

For peer review only

- whether continuous improvement methods are used to refine the data collection approach, based on performance data
- Types of comparison
- Types of outcome measures
- Outcomes: response rate, completeness, equivalence, time, and costs - as described above. We will extract all qualitative text provided on acceptability.

Risk of bias assessment for included studies

We will use the Cochrane RoB2 tool for randomised studies[30 31] and RoB-I for non-randomised studies.[32] Two reviewers will independently assess risk of bias. The review authors will resolve disagreements through a consensus-based decision, or if necessary, discussion with a third reviewer.

The risk of bias for each outcome across individual studies will be summarised as a narrative statement and supported by a risk of bias table. A review-level narrative summary of the risk of bias will also be provided.

Contacting study authors

We will contact study authors to request additional information and primary data where any aspect precludes the assessment of eligibility or inclusion in the data synthesis.

Strategy for data synthesis

If data are available, we will pool effect estimates using a random-effects model.[33]. However, we anticipate heterogeneity in study design, interventions and outcomes and therefore plan to use a narrative synthesis without meta-analysis approach, following the SWiM reporting guidelines from Campbell and colleagues.[34] We will stratify the synthesis by intervention type and outcome.

Assessment of heterogeneity

We will assess heterogeneity by considering study design, interventions, outcomes.

Analysis of subgroups or subsets

We will assess whether response rates for each modality vary according to age, sex, urban/rural, ethnicity, and marital status where baseline data on the distribution of these characteristics within the general population are available.

We will perform a secondary analysis examining whether findings differ between high-income and low- and middle-income countries.

We will repeat comparisons between data collection approaches using the subset of included studies that only examine one approach.

Meta-biases

It is unlikely that we will be able to assess publication bias because it would require meta-analyses of ten or more studies, but if we do have such an analysis we will create a funnel plot.[35] Selective outcome reporting will be assessed by comparing protocols (where available) with published reports.

For peer review only

Assessment of certainty of evidence
Where possible, the GRADE criteria will be used to assess the certainty of the primary outcomes.[36 37] One review author will collate the evidence for each primary outcome and suggest initial ratings. These will be deliberated by a team of review authors who will reach a joint decision for each outcome. For RCTs evidence will be assumed to be high certainty and then will be downgraded due to risk of bias, inconsistency of results, indirectness of evidence, imprecision, publication bias. For observational studies, evidence starts at low-certainty but can be upgraded if there is a large effect, dose-response, gradient, or plausible confounding that decreases the magnitude of effect.

Conclusion

Gathering data on socioeconomic status (SES) is a prerequisite for any health programme that aims to assess and improve the equitable distribution of its outcomes. The aim of this review is to compare three different modalities for gathering SES data in terms of methodological performance, costs, and perceived benefits. To our knowledge, there has been no previous synthesis of this literature. The findings of this review will be used to help eye health screening programmes collect SES data with a view to optimising the equitable distribution of programme benefits. We believe the findings of this review will be useful for programme managers and policymakers working in a wide range of fields.

Ethics and dissemination

Ethical approval is not required, as our review will only include published and publicly accessible data.

We will publish our findings in an open-access, peer-reviewed journal and develop an accessible summary of the results for website posting and stakeholder meetings. Data generated from this review will be made available upon reasonable request.

Authors' contributions
LA and IG drafted and revised the protocol with suggestions from all other authors who reviewed the protocol and provided feedback on the draft. IG constructed the search. All authors read and approved the final protocol.

Funding
This work was supported by the Wellcome Trust and NIHR grant number 215633/Z/19/Z.

The funders had no role in the development of the protocol and will not play any role in the execution of the systematic review.

Competing interests

For peer review only

None declared.

Patient consent for publication

Not required.

For peer review only

Appendix: Search strategies

MEDLINE

Ovid MEDLINE(R) and Epub Ahead of Print, In-Process, In-Data-Review & Other Non-Indexed Citations and Daily

1. Telephone/
2. (telephone$ or phone$).tw.
3. ((voice or phone) adj1 call$).tw.
4. (phone adj2 interview$).tw.
5. Cell Phones/
6. Smartphone/
7. (phone$ adj1 (smart or cell)).tw.
8. (smartphone$ or cellphone$).tw.
9. (mobile adj2 (phone$ or device$)).tw.
10. or/1-9
11. Text Messaging/
12. (text or texts or texting).tw.
13. MMS.tw.
14. SMS.tw.
15. short message service.tw.
16. multimedia message service.tw.
17. (automated adj2 (telephone$ or text$ or message$ or questionnaire$)).tw.
18. (telephone adj1 administered adj1 questionnaire$).tw.
19. or/11-18
20. Interviews as Topic/
21. Patient Health Questionnaire/
22. Self Report/
23. (in adj1 person$).tw.
24. (in adj1 person$ adj4 (interview$ or survey$ or question$)).tw.
25. (face adj2 face adj4 (interview$ or survey$ or question$)).tw.
26. (face-to-face adj4 (interview$ or survey$ or question$)).tw.
27. or/20-26
28. 10 and 19
29. 10 and 27
30. 19 and 27
31. 10 and 19 and 27
32. 28 or 29 or 30 or 31
33. Vulnerable populations/ or socioeconomic factors/ or poverty/ or social class/ or Healthcare Disparities/ or Health Status Disparities/ or Poverty areas/ or Urban population/
34. (equit$ or inequit$ or inequalit$ or disparit$ or equality).tw.
35. (ethnic$ or race or racial$ or caste$).tw.
36. ((social$ or socio-economic or socioeconomic or economic or structural or material) adj3 (advantage$ or disadvantage$ or exclude$ or exclusion or include$ or inclusion or status or position or gradient$ or hierarch$ or class$ or determinant$)).tw.

For peer review only

37. (health adj3 (gap$ or gradient$ or hierarch$)).tw.
38. exp education/ or educational status/ or employment/ or income/ or occupations/ or social conditions/
39. (SES or SEP or sociodemographic$ or socio-demographic$ or demographic$ or income or wealth$ or poverty or affluen$).tw.
40. (educat$ adj3 (level$ or attain$ or status or well or better)).tw.
41. (occupation or unemploy$).tw.
42. (home owner$ or tenure).tw.
43. (household adj2 (income or wealth or status)).tw.
44. ((well or better or worse) adj2 off).tw.
45. or/33-43
46. Community Health Planning/
47. Community Health Services/
48. Community Health Nursing/
49. National Health Programs/
50. State Medicine/
51. Regional Health Planning/
52. Health Planning/
53. Health Plan Implementation/
54. Health Planning Guidelines/
55. Health Care Reform/
56. Health Resources/
57. Health Priorities/
58. Health Services Research/
59. "health services needs and demand"/
60. Needs Assessment/
61. State Health Plans/
62. Regional Health Planning/
63. Primary Health Care/
64. Health Services, Indigenous/
65. Rural Health Services/
66. Mobile Health Units/
67. randomized controlled trial/ or controlled clinical trials as topic/ or randomized controlled trials as topic/
68. (randomized or randomised or randomly or RCT).tw.
69. outcome assessment, health care/
70. comparative study/ or evaluation studies/ or meta-analysis/ or multicenter study/ or "systematic review"/ or validation studies/
71. epidemiologic studies/ or follow-up studies/ or longitudinal studies/ or prospective studies/ or controlled before-after studies/
72. or/46-71
73. 32 and 45 and 72
74. limit 73 to yr="1999 -Current"

For peer review only

Cochrane Library

#1 MeSH descriptor: [Telephone] this term only
#2 telephone* or phone*
#3 (voice or phone) near/1 call*
#4 phone near/2 interview*
#5 MeSH descriptor: [Cell Phone] this term only
#6 MeSH descriptor: [Smartphone] this term only
#7 phone* near/1 (smart or cell)
#8 smartphone* or cellphone*
#9 mobile near/2 (phone* or device*)
#10 #1 or #2 or #3 or #4 or #5 or #6 or #7 or #8 or #9
#11 MeSH descriptor: [Text Messaging] this term only
#12 text or texts or texting
#13 MMS or SMS
#14 "multimedia message service"
#15 "short message service"
#16 automated near/2 (telephone* or text* or message* or questionnaire*)
#17 telephone near/1 administered near/1 questionnaire*
#18 #11 or #12 or #13 or #14 or #15 or #16 or #17
#19 MeSH descriptor: [Interviews as Topic] this term only
#20 MeSH descriptor: [Patient Health Questionnaire] this term only
#21 MeSH descriptor: [Self Report] this term only
#22 in near/1 person*
#23 (in near/1 person* near/4 (interview* or survey* or question*))
#24 (face near/2 face near/4 (interview* or survey* or question*))
#25 (face-to-face near/4 (interview* or survey* or question*))
#26 #19 or #20 or #21 or #22 or #23 or #24 or #25
#27 #10 and #18
#28 #10 and #26
#29 #18 and #26
#30 #10 and #18 and #26
#31 #27 or #28 or #29 or #30
#32 MeSH descriptor: [Socioeconomic Factors] this term only
#33 MeSH descriptor: [Poverty] this term only
#34 MeSH descriptor: [Social Class] this term only
#35 MeSH descriptor: [Vulnerable Populations] this term only
#36 MeSH descriptor: [Healthcare Disparities] this term only
#37 MeSH descriptor: [Health Status Disparities] this term only
#38 MeSH descriptor: [Poverty Areas] this term only
#39 MeSH descriptor: [Urban Population] this term only
#40 equit* or inequit* or inequalit* or disparit* or equality
#41 ethnic* or race or racial* or caste*

For peer review only

#42 (social* or socio-economic or socioeconomic or economic or structural or material) near/3 (advantage* or disadvantage* or exclude* or exclusion or include* or inclusion or status or position or gradient* or hierarch* or class* or determinant*)
#43 health near/3 (gap* or gradient* or hierarch*)
#44 MeSH descriptor: [Education] explode all trees
#45 MeSH descriptor: [Educational Status] this term only
#46 MeSH descriptor: [Employment] this term only
#47 MeSH descriptor: [Income] this term only
#48 MeSH descriptor: [Occupations] this term only
#49 MeSH descriptor: [Social Conditions] this term only
#50 SES or SEP or sociodemographic* or socio-demographic* or income or wealth* or poverty or affluen*
#51 educat* near/3 (level* or attain* or status or well or better)
#52 occupation or unemploy*
#53 home owner* or tenure
#54 household near/2 (income or wealth or status)
#55 (well or better or worse) near/2 off
#56 #32 or #33 or #34 or #35 or #36 or #37 or #38 or #39 or #40 or #41 or #42 or #43 or #44 or #45 or #46 or #47 or #48 or #49 or #50 or #51 or #52 or #53 or #54 or #55
#57 MeSH descriptor: [Community Health Planning] this term only
#58 MeSH descriptor: [Community Health Services] this term only
#59 MeSH descriptor: [Community Health Nursing] explode all trees
#60 MeSH descriptor: [National Health Programs] this term only
#61 MeSH descriptor: [State Medicine] explode all trees
#62 MeSH descriptor: [Regional Health Planning] this term only
#63 MeSH descriptor: [Health Planning] this term only
#64 MeSH descriptor: [Health Plan Implementation] this term only
#65 MeSH descriptor: [Health Planning Guidelines] this term only
#66 MeSH descriptor: [Health Care Reform] this term only
#67 MeSH descriptor: [Health Resources] this term only
#68 MeSH descriptor: [Health Priorities] this term only
#69 MeSH descriptor: [Health Services Research] this term only
#70 MeSH descriptor: [Health Services Needs and Demand] this term only
#71 MeSH descriptor: [Needs Assessment] this term only
#72 MeSH descriptor: [State Health Plans] this term only
#73 MeSH descriptor: [Regional Health Planning] this term only
#74 MeSH descriptor: [Primary Health Care] this term only
#75 MeSH descriptor: [Health Services, Indigenous] this term only
#76 MeSH descriptor: [Rural Health Services] this term only
#77 MeSH descriptor: [Mobile Health Units] this term only
#78 #57 or #58 or #59 or #60 or #61 or #62 or #63 or #64 or #65 or #66 or #67 or #68 or #69 or #70 or #71 or #72 or #73 or #74 or #75 or #76 or #77
#79 #31 and #56 and #78 with Publication Year from 1999 to 2021, in Trials

Embase

For peer review only

1. telephone/
2. telephone interview/
3. (telephone$ or phone$).tw.
4. ((voice or phone) adj1 call$).tw.
5. (phone adj2 interview$).tw.
6. mobile phone/
7. smartphone/
8. (phone$ adj1 (smart or cell)).tw.
9. (smartphone$ or cellphone$).tw.
10. (mobile adj2 (phone$ or device$)).tw.
11. or/1-10
12. text messaging/
13. (text or texts or texting).tw.
14. MMS.tw.
15. SMS.tw.
16. multimedia message service.tw.
17. short message service.tw.
18. (automated adj2 (telephone$ or text$ or message$ or questionnaire$)).tw.
19. (telephone adj1 administered adj1 questionnaire$).tw.
20. or/12-19
21. interview/
22. (in adj1 person$).tw.
23. (in adj1 person$ adj4 (interview$ or survey$ or question$)).tw.
24. (face adj2 face adj4 (interview$ or survey$ or question$)).tw.
25. (face-to-face adj4 (interview$ or survey$ or question$)).tw.
26. or/21-25
27. 11 and 20
28. 11 and 26
29. 20 and 26
30. 11 and 20 and 26
31. 27 or 28 or 29 or 30
32. socioeconomics/
33. poverty/
34. social status/
35. social class/
36. vulnerable population/
37. health care disparity/
38. health disparity/
39. urban population/
40. (equit$ or inequit$ or inequalit$ or disparit$ or equality).tw.
41. (ethnic$ or race or racial$ or caste$).tw.
42. ((social$ or socio-economic or socioeconomic or economic or structural or material) adj3 (advantage$ or disadvantage$ or exclude$ or exclusion or include$ or inclusion or status or position or gradient$ or hierarch$ or class$ or determinant$)).tw.

For peer review only

43. (health adj3 (gap$ or gradient$ or hierarch$)).tw.
44. education/
45. educational status/
46. employment/
47. employment status/
48. unemployment/
49. household income/ or family income/ or income/
50. occupation/
51. (SES or SEP or sociodemographic$ or socio-demographic$ or demographic$ or income or wealth$ or poverty or affluen$).tw.
52. (educat$ adj3 (level$ or attain$ or status or well or better)).tw.
53. (occupation or unemploy$).tw.
54. (home owner$ or tenure).tw.
55. (household adj2 (income or wealth or status)).tw.
56. ((well or better or worse) adj2 off).tw.
57. or/32-56
58. public health/
59. health care planning/
60. community care/
61. community health nursing/
62. national health service/
63. health care policy/
64. health services research/
65. health service/
66. primary health care/
67. indigenous health care/
68. rural health care/
69. randomized controlled trial/ or controlled clinical trial/ or "randomized controlled trial (topic)"/
70. (randomized or randomised or randomly or RCT).tw.
71. outcome assessment/
72. comparative study/
73. evaluation study/
74. "systematic review"/ or "systematic review (topic)"/ or meta analysis/
75. epidemiology/
76. prospective study/
77. longitudinal study/
78. follow up/
79. or/58-78
80. 31 and 57 and 79
81. limit 80 to yr="1999 -Current"

Global Health

For peer review only

1. mobile telephones/ or telephones/
2. (telephone$ or phone$).tw.
3. ((voice or phone) adj1 call$).tw.
4. (phone adj2 interview$).tw.
5. (phone$ adj1 (smart or cell)).tw.
6. (smartphone$ or cellphone$).tw.
7. (mobile adj2 (phone$ or device$)).tw.
8. or/1-7
9. (text or texts or texting).tw.
10. (MMS or SMS).tw.
11. multimedia message service.tw.
12. short message service.tw.
13. (automated adj2 (telephone$ or text$ or message$ or questionnaire$)).tw.
14. (telephone adj1 administered adj1 questionnaire$).tw.
15. or/9-14
16. interviews/
17. (in adj1 person).tw.
18. (in adj1 person adj4 (interview$ or survey$ or question$)).tw.
19. (face adj2 face adj4 (interview$ or survey$ or question$)).tw.
20. (face-to-face adj4 (interview$ or survey$ or question$)).tw.
21. or/16-20
22. 7 and 15
23. 7 and 21
24. 15 and 21
25. 7 and 15 and 21
26. 22 or 23 or 24 or 25
27. socioeconomic status/ or socioeconomics/
28. poverty/
29. exp social classes/ or caste/ or social inequalities/ or social mobility/
30. urban population/
31. (equit$ or inequit$ or inequalit$ or disparit$ or equality).tw.
32. (ethnic$ or race or racial$ or caste$).tw.
33. ((social$ or socio-economic or socioeconomic or economic or structural or material) adj3 (advantage$ or disadvantage$ or exclude$ or exclusion or include$ or inclusion or status or position or gradient$ or hierarch$ or class$ or determinant$)).tw.
34. (health adj3 (gap$ or gradient$ or hierarch$)).tw.
35. education/
36. employment/
37. occupations/
38. income/ or household income/
39. living conditions/
40. (SES or SEP or sociodemographic$ or socio-demographic$ or demographic$ or income or wealth$ or poverty or affluen$).tw.
41. (educat$ adj3 (level$ or attain$ or status or well or better)).tw.
42. (occupation or unemploy$).tw.

For peer review only

43. (home owner$ or tenure).tw.
44. (household adj2 (income or wealth or status)).tw.
45. ((well or better or worse) adj2 off).tw.
46. or/27-45
47. 26 and 46
48. limit 47 to yr="1999 -Current"

ClinicalTrials.gov

Search 1
socioeconomic AND (telephone OR phone) AND (interview OR face-to-face OR in-person) AND community | Interventional Studies

Search 2
socioeconomic AND (telephone OR phone) AND (text OR SMS OR MMS) AND community | Interventional Studies

Search 3
socioeconomic AND (text OR SMS OR MMS) AND (interview OR face-to-face OR in-person) AND community | Interventional Studies

WHO ICTRP

Search 1
socioeconomic AND telephone AND interview AND community

Search 2
socioeconomic AND telephone AND text AND community

Search 3
socioeconomic AND text AND interview AND community

OpenGrey

socioeconomic AND (telephone OR phone OR text OR interview OR face-to-face OR in-person) AND community

References

[1] Marmot M. Social determinants of health inequalities. The Lancet. 2005 Mar 19;365(9464):1099-104.
[2] Hart JT. The inverse care law. The Lancet. 1971 Feb 27;297(7696):405-12.

For peer review only - http://bmjopen.bmj.com/site/about/guidelines.xhtml

---

[3] WHO. Constitution of the World HEalth Organization. 1948. Available at: https://apps.who.int/gb/bd/PDF/bd47/EN/constitution-en.pdf?ua=1 [Accessed 11/05/2021]

[4] United Nations General Assembly. The Sustainable Development Goals. Available at: https://www.who.int/topics/sustainable-development-goals/targets/en/ [Accessed 11/05/2021]

[5] Ng M, Fullman N, Dieleman JL, Flaxman AD, Murray CJ, Lim SS. Effective coverage: a metric for monitoring universal health coverage. PLoS Med. 2014 Sep 22;11(9):e1001730.

[6] Rodney AM, Hill PS. Achieving equity within universal health coverage: a narrative review of progress and resources for measuring success. International journal for equity in health. 2014 Dec;13(1):1-8.

[7] Guo S, Carvajal-Aguirre L, Victora CG, Barros AJ, Wehrmeister FC, Vidaletti LP, Gupta G, Matin MZ, Rutter P. Equitable coverage? The roles of the private and public sectors in providing maternal, newborn and child health interventions in South Asia. BMJ global health. 2019 Aug 1;4(4):e001495.

[8] Carey G, Crammond B, De Leeuw E. Towards health equity: a framework for the application of proportionate universalism. International journal for equity in health. 2015 Dec;14(1):1-8.

[9] Burton MJ, Ramke J, Marques AP, Bourne RR, Congdon N, Jones I, Tong BA, Arunga S, Bachani D, Bascaran C, Bastawrous A. The Lancet global health Commission on global eye health: vision beyond 2020. The Lancet Global Health. 2021 Apr 1;9(4):e489-551.

[10] Allen LN, Smith RW, Simmons-Jones F, Roberts N, Honney R, Currie J. Addressing social determinants of noncommunicable diseases in primary care: a systematic review. Bulletin of the World Health Organization. 2020 Nov 1;98(11):754.

[11] Peek Vision. Available at: https://www.peekvision.org/ [Accessed 14/05/2021].

[12] The King's Fund. Community health services explained. Available at: https://www.kingsfund.org.uk/publications/community-health-services-explained [Accessed 13/05/2021].

[13] American Psychological Association. Socioeconomic status. Available at: https://www.apa.org/topics/socioeconomic-status [Accessed 13/05/2021].

[14] WHO. Closing the gap in a generation: The commission on the social determinants of health. Available at: https://www.who.int/publications/i/item/WHO-IER-CSDH-08.1 [Accessed 13/05/2021].

[15] World Health Organization. Social determinants of health. Available at: https://www.who.int/news-room/q-a-detail/social-determinants-of-health-key-concepts [Accessed 13/05/2021].

[16] World Health Organization. Social determinants of health: the solid facts. World Health Organization. Regional Office for Europe; 2003.

[17] Marmot M. Social determinants of health inequalities. The Lancet. 2005 Mar 19;365(9464):1099-104.

[18] American Psychological Association. Socioeconomic status. Available at: https://www.apa.org/topics/socioeconomic-status [Accessed 13/05/2021].

[19] Preston SH. The changing relation between mortality and level of economic development. Population studies. 1975 Jul 1;29(2):231-48.

[20] Preston SH. The changing relation between mortality and level of economic development. Int J Epidemiol. 2007; 36: 484-490

[21] Bloom DE, Canning D. Commentary: The Preston Curve 30 years on: still sparking fires. Int J Epidemiol. 2007 Jun 1;36(3):498-9.

[22] World Bank. Why use GNI per capita to classify economies into income groupings? Available at: https://datahelpdesk.worldbank.org/knowledgebase/articles/378831-why-use-gni-per-capita-to-classify-economies-into [Accessed 24/06/2021].

[23] World Bank. Country and lending groups: fiscal year 2021. Available at: https://datahelpdesk.worldbank.org/knowledgebase/articles/906519-world-bank-country-and-lending-groups [Accessed 13/05/2021].

[24] World Bank. The Altas method - detailed methodology. Available at: https://datahelpdesk.worldbank.org/knowledgebase/articles/378832-what-is-the-world-bank-atlas-method [Accessed 13/05/2021].

[25] Shamseer L, Moher D, Clarke M, Ghersi D, Liberati A, Petticrew M, Shekelle P, Stewart L, PRISMA-P Group. Preferred reporting items for systematic review and meta-analysis protocols (PRISMA-P) 2015: elaboration and explanation. BMJ. 2015 Jan 2;349(jan02 1):g7647.

For peer review only

[26] Belisario JS, Jamsek J, Huckvale K, O'Donoghue J, Morrison CP, Car J. Comparison of self-administered survey questionnaire responses collected using mobile apps versus other methods. Cochrane database of systematic reviews. 2015(7).
[27] Gwaltney CJ, Shields AL, Shiffman S. Equivalence of electronic and paper-and-pencil administration of patient-reported outcome measures: a meta-analytic review. Value in Health 2008;11(2):322-33.
[28] Page et al. The PRISMA 2020 statement: an updated guideline for reporting systematic reviews BMJ 2021; 372.
[29] Cochrane. Template collection form for RCTs and non RCTs. Available at: https://training.cochrane.org/sites/training.cochrane.org/files/public/uploads/resources/downloadable_resources/English/Collecting%20data%20-%20form%20for%20RCTs%20and%20non-RCTs.doc
[30] Risk of Bias tool. Available at: https://www.riskofbias.info/. [Accessed 18/05/2021].
[31] Sterne JAC, Savović J, Page MJ, Elbers RG, Blencowe NS, Boutron I, Cates CJ, Cheng H-Y, Corbett MS, Eldridge SM, Hernán MA, Hopewell S, Hróbjartsson A, Junqueira DR, Jüni P, Kirkham JJ, Lasserson T, Li T, McAleenan A, Reeves BC, Shepperd S, Shrier I, Stewart LA, Tilling K, White IR, Whiting PF, Higgins JPT. RoB 2: a revised tool for assessing risk of bias in randomised trials. BMJ 2019; 366: l4898.
[32]Sterne JAC, Hernán MA, Reeves BC, Savović J, Berkman ND, Viswanathan M, Henry D, Altman DG, Ansari MT, Boutron I, Carpenter JR, Chan AW, Churchill R, Deeks JJ, Hróbjartsson A, Kirkham J, Jüni P, Loke YK, Pigott TD, Ramsay CR, Regidor D, Rothstein HR, Sandhu L, Santaguida PL, Schünemann HJ, Shea B, Shrier I, Tugwell P, Turner L, Valentine JC, Waddington H, Waters E, Wells GA, Whiting PF, Higgins JPT. ROBINS-I: a tool for assessing risk of bias in non-randomized studies of interventions. BMJ 2016; 355; i4919; doi: 10.1136/bmj.i4919.
[33] Deeks JJ, Higgins JPT, Altman DG. Cochrane Handbook Chapter 10: Analysing data and undertaking meta-analyses. Available at: https://training.cochrane.org/handbook/current/chapter-10 [Accessed 18/05/2021].
[34] Campbell M, McKenzie JE, Sowden A, Katikireddi SV, Brennan SE, Ellis S, Hartmann-Boyce J, Ryan R, Shepperd S, Thomas J, Welch V, Thomson H. Synthesis without meta-analysis (SWiM) in systematic reviews: reporting guideline BMJ 2020;368:l6890 http://dx.doi.org/10.1136/bmj.l6890
[35] Page MJ, Higgins JPT, Sterne JAC. Cochrane Handbook Chapter 13: Assessing risk of bias due to missing results in a synthesis. Available at: https://training.cochrane.org/handbook/current/chapter-13 [Accessed 18/05/2021].
[36] Murad MH, Mustafa RA, Schünemann HJ, Sultan S, Santesso N. Rating the certainty in evidence in the absence of a single estimate of effect. BMJ Evidence-Based Medicine. 2017 Jun 1;22(3):85-7.
[37] GRADE. The GRADE handbook. Available at: https://gdt.gradepro.org/app/handbook/handbook.html [Accessed 18/05/2021].

For peer review only

PRISMA-P (Preferred Reporting Items for Systematic review and Meta-Analysis Protocols) 2015 checklist: recommended items to address in a systematic review protocol*

Section and topic	Item No	Checklist item
**ADMINISTRATIVE INFORMATION**		
Title:		
Identification	1a	Identify the report as a protocol of a systematic review
Update	1b	If the protocol is for an update of a previous systematic review, identify as such
Registration	2	If registered, provide the name of the registry (such as PROSPERO) and registration number
Authors:		
Contact	3a	Provide name, institutional affiliation, e-mail address of all protocol authors; provide physical mailing address of corresponding author
Contributions	3b	Describe contributions of protocol authors and identify the guarantor of the review
Amendments	4	If the protocol represents an amendment of a previously completed or published protocol, identify as such and list changes; otherwise, state plan for documenting important protocol amendments
Support:		
Sources	5a	Indicate sources of financial or other support for the review
Sponsor	5b	Provide name for the review funder and/or sponsor
Role of sponsor or funder	5c	Describe roles of funder(s), sponsor(s), and/or institution(s), if any, in developing the protocol
**INTRODUCTION**		
Rationale	6	Describe the rationale for the review in the context of what is already known
Objectives	7	Provide an explicit statement of the question(s) the review will address with reference to participants, interventions, comparators, and outcomes (PICO)
**METHODS**		
Eligibility criteria	8	Specify the study characteristics (such as PICO, study design, setting, time frame) and report characteristics (such as years considered, language, publication status) to be used as criteria for eligibility for the review
Information sources	9	Describe all intended information sources (such as electronic databases, contact with study authors, trial registers or other grey literature sources) with planned dates of coverage
Search strategy	10	Present draft of search strategy to be used for at least one electronic database, including planned limits, such that it could be repeated
Study records:		
Data management	11a	Describe the mechanism(s) that will be used to manage records and data throughout the review

For peer review only

Selection process	11b	State the process that will be used for selecting studies (such as two independent reviewers) through each phase of the review (that is, screening, eligibility and inclusion in meta-analysis)
Data collection process	11c	Describe planned method of extracting data from reports (such as piloting forms, done independently, in duplicate), any processes for obtaining and confirming data from investigators
Data items	12	List and define all variables for which data will be sought (such as PICO items, funding sources), any pre-planned data assumptions and simplifications
Outcomes and prioritization	13	List and define all outcomes for which data will be sought, including prioritization of main and additional outcomes, with rationale
Risk of bias in individual studies	14	Describe anticipated methods for assessing risk of bias of individual studies, including whether this will be done at the outcome or study level, or both; state how this information will be used in data synthesis
Data synthesis	15a	Describe criteria under which study data will be quantitatively synthesised
	15b	If data are appropriate for quantitative synthesis, describe planned summary measures, methods of handling data and methods of combining data from studies, including any planned exploration of consistency (such as I^2, Kendall's τ)
	15c	Describe any proposed additional analyses (such as sensitivity or subgroup analyses, meta-regression)
	15d	If quantitative synthesis is not appropriate, describe the type of summary planned
Meta-bias(es)	16	Specify any planned assessment of meta-bias(es) (such as publication bias across studies, selective reporting within studies)
Confidence in cumulative evidence	17	Describe how the strength of the body of evidence will be assessed (such as GRADE)

*** It is strongly recommended that this checklist be read in conjunction with the PRISMA-P Explanation and Elaboration (cite when available) for important clarification on the items. Amendments to a review protocol should be tracked and dated. The copyright for PRISMA-P (including checklist) is held by the PRISMA-P Group and is distributed under a Creative Commons Attribution Licence 4.0.**

From: Shamseer L, Moher D, Clarke M, Ghersi D, Liberati A, Petticrew M, Shekelle P, Stewart L, PRISMA-P Group. Preferred reporting items for systematic review and meta-analysis protocols (PRISMA-P) 2015: elaboration and explanation. BMJ. 2015 Jan 2;349(jan02 1):g7647.

For peer review only

BMJ Open

Performance and resource requirements of in-person vs voice call vs automated telephone-based socioeconomic data collection modalities for community-based health programmes: a systematic review protocol

Journal:	*BMJ Open*
Manuscript ID	bmjopen-2021-057410.R1
Article Type:	Protocol
Date Submitted by the Author:	03-Mar-2022
Complete List of Authors:	Allen, Luke; London School of Hygiene & Tropical Medicine, Department of Clinical Research<br>Mackinnon, Shona; University of Glasgow<br>Gordon, Iris; London School of Hygiene & Tropical Medicine, Department of Clinical Research<br>Blane, David; University of Glasgow<br>Marques, Ana Patricia; London School of Hygiene and Tropical Medicine International Centre for Eye Health,<br>Gichuhi, Stephen; University of Nairobi<br>Mwangi, Alice; Operation Eyesight<br>Burton, Matthew J; London School of Hygiene & Tropical Medicine, Department of Clinical Research<br>Bolster, Nigel; Peek Vision<br>Macleod, David; London School of Hygiene & Tropical Medicine<br>Kim, Min; London School of Hygiene and Tropical Medicine Faculty of Infectious and Tropical Diseases<br>Ramke, Jacqueline; London School of Hygiene & Tropical Medicine, Department of Clinical Research<br>Bastawrous, Andrew; London School of Hygiene & Tropical Medicine
**Primary Subject Heading**:	Public health
Secondary Subject Heading:	Health services research
Keywords:	PRIMARY CARE, PUBLIC HEALTH, Epidemiology < TROPICAL MEDICINE

SCHOLARONE™
Manuscripts

Performance and resource requirements of in-person vs voice call vs automated telephone-based socioeconomic data collection modalities for community-based health programmes: a systematic review protocol

Authors

Dr Luke N Allen (corresponding author)
International Centre for Eye Health, Department of Clinical Research, London School of Hygiene & Tropical Medicine, Keppel St, London WC1E 7HT
drlukeallen@gmail.com
ORCID ID: 0000-0003-2750-3575

Dr Shona Mackinnon
NHS Education for Scotland
shonamackinnon@doctors.org.uk

Ms Iris Gordon
International Centre for Eye Health, Department of Clinical Research, London School of Hygiene & Tropical Medicine, Keppel St, London WC1E 7HT
ORCID: 0001-8143-8132
iris.gordon@lshtm.ac.uk

Dr David Blane
Institute of Health and Wellbeing, University of Glasgow
David.Blane@glasgow.ac.uk
ORCID ID: 0000-0002-3872-3621

Dr Ana Patricia Marques
International Centre for Eye Health, Department of Clinical Research, London School of Hygiene & Tropical Medicine, Keppel St, London WC1E 7HT
Patricia.Marques@lshtm.ac.uk
ORCID ID: 0000-0001-8242-7021

Dr Stephen Gichuhi
MBChB, M.Med (Ophth), MBA, MSc (Epid), PhD, FCOphth(ECSA)
Senior Lecturer & Consultant Ophthalmologist, Chairman, Department of Ophthalmology, University of Nairobi
stephen.gichuhi@lshtm.ac.uk

Ms Alice Mwangi
Operation Eyesight
mwangia@operationeyesight.com

For peer review only - http://bmjopen.bmj.com/site/about/guidelines.xhtml

Prof Matthew J. Burton
International Centre for Eye Health, Department of Clinical Research, London School of Hygiene & Tropical Medicine, Keppel St, London WC1E 7HT
matthew.burton@lshtm.ac.uk

Dr Nigel Bolster
International Centre for Eye Health, Department of Clinical Research, London School of Hygiene & Tropical Medicine, Keppel St, London WC1E 7HT

Peek Vision, 90a High Street, Berkhamsted, Hertfordshire, England HP4 2BL
nigel@peekvision.org
ORCID: 0000-0001-6607-1723

Dr David Macleod
International Statistics & Epidemiology Group, Department of Infectious Disease Epidemiology, London School of Hygiene & Tropical Medicine, Keppel St, London WC1E 7HT
david.macleod@lshtm.ac.uk

Ms Min Kim
International Centre for Eye Health, Department of Clinical Research, London School of Hygiene & Tropical Medicine, Keppel St, London WC1E 7HT
min.kim@lshtm.ac.uk

Assoc Prof Jacqueline Ramke
International Centre for Eye Health, Department of Clinical Research, London School of Hygiene & Tropical Medicine, Keppel St, London WC1E 7HT
School of Optometry and Vision Science, University of Auckland, Auckland, New Zealand
Jacqueline.Ramke@lshtm.ac.uk

Assoc Prof Andrew Bastawrous
International Centre for Eye Health, Department of Clinical Research, London School of Hygiene & Tropical Medicine, Keppel St, London WC1E 7HT
Andrew.bastawrous@lshtm.ac.uk

Abstract

Introduction

Gathering data on socioeconomic status (SES) is a prerequisite for any health programme that aims to assess and improve the equitable distribution of its outcomes. Many different modalities can be used to collect SES data, ranging from (1) face-to-face elicitation, to (2) telephone-administered questionnaires, to (3) automated text message-based systems. The relative costs and perceived benefits to patients and providers of these different data collection approaches is unknown. This protocol is for a systematic review that aims to compare the resource requirements, performance characteristics, and acceptability to participants and service providers of these three approaches to collect SES data from those enrolled in health programmes.

Methods and analysis

An information specialist will conduct searches on the Cochrane Library, MEDLINE, Embase, Global Health, Clinicaltrials.gov, the WHO ICTRP and OpenGrey. All databases will be searched from 1999 to present with no language limits used. We will also search Google Scholar and check the reference lists of relevant articles for further potentially eligible studies. Any empirical study design will be eligible if it compares two or more modalities to elicit SES data from the following three; in-person, voice call, or automated phone-based systems. Two reviewers will independently screen titles, abstracts and full-text articles; and complete data extraction. For each study we will extract data on the modality characteristics, primary outcomes (response rate, completeness, and equivalence) and secondary outcomes (time, costs, and acceptability to patients and providers). We will synthesise findings thematically without meta-analysis.

Ethics and dissemination

Ethical approval is not required, as our review will include published and publicly accessible data. This review is part of a project to improve equitable access to eye care services in low- and middle-income countries. However, the findings will be useful to policymakers and programme managers in a range of health- and non-health settings. We will publish our findings in a peer-reviewed journal and develop an accessible summary of results for website posting and stakeholder meetings.

Registration

This review is registered with PROSPERO CRD42021251959

Strengths and limitations of this study

- As far as we are aware, this review will be the first to directly compare three commonly used data collection modalities for the collection of SES data.
- The review will be comprehensive, covering published and grey literature in any language.
- This review will be robust, using independent dual review at every stage, and following best-practice guidelines.
- There may only be a small number of articles in the literature that compare the different modalities head-to-head and provide data on the outcomes of interest.

For peer review only

Introduction

Rationale

Inequalities in health are pervasive and stubbornly persistent. Individuals with lower levels of income, education, and social status tend to experience the worst health outcomes irrespective of where they are in the world.[1] In 1971 Julian Tudor Hart observed that the availability of good medical care tends to vary inversely with the need for it in the population served.[2] This inverse care law manifests in the majority of global health and development programmes where individuals with the lowest socioeconomic status (SES) tend to face the highest barriers in accessing care and are the least likely to attain good outcomes.

Recognising marked inter- and intra-national disparities in health outcomes, the World Health Organisation (WHO) was constituted in 1948 with the mandate of advancing 'health for all'.[3] The contemporary manifestation of this mission is encapsulated in the concept of Universal Health Coverage (and Sustainable Development Goal target 3.8[4]) which seeks to extend coverage to disenfranchised groups. Emerging emphases on attaining *effective* coverage,[5] and *equitable* coverage[6 7] seek to shift the success criteria from supply-side provision of services to demand-side receipt of effective services according to need. These trends are underpinned by the principle of 'proportionate universalism': seeking to improve the health of all, with the greatest gains experienced by those with the greatest needs.[8] There is also an increasing interest in understanding the distribution of programme benefits across socio-demographic groups - for instance women, those living in rural locations, and those living in conditions of poverty.[9]

All attempts to boost equity in service provision are predicated on adequate collection and analysis of sociodemographic data. Previous work has demonstrated that sociodemographic data can be collected using a variety of modalities in the community setting including in-person, telephone voice calls, and using automated telephone-based systems[10] (Box 1). However, as far as we are aware, the relative costs and benefits of the different modalities have not been studied, including the skills, equipment, time and financial resources required and acceptability to data collectors and service beneficiaries.

This review aims to answer the research question 'how do three common SES data collection modalities compare in terms of performance characteristics, resource requirements and acceptability to participants and service providers?' Selecting an appropriate and cost-effective modality is an important first step towards advancing equitable effective service coverage.

The findings of this review will directly inform the development of school and community-based eye health screening programmes that operates in several low- and middle-income countries (LMICs) including Botswana, Ethiopia, Kenya, Nepal, Pakistan, Tanzania, Uganda and Zimbabwe.[11] However, the collection of SES data is relevant for a much wider range of global health programmes, as well as non-health programmes aimed at improving educational, agricultural, gender equity, and economic outcomes, among others.

Descriptions of the interventions

For peer review only - http://bmjopen.bmj.com/site/about/guidelines.xhtml

Three different modalities for SES data collection constitute the interventions of interest for this review: in-person; voice call; and automated telephone data collection. Box 1 provides the definition for each.

Box 1: Definitions of the three data collection approaches used in this review

In-person data collection includes any form of exchange between a programme implementer and a participant or their responsible guardian, whereby the programme implementer asks predefined questions to ascertain the participants' socioeconomic status and a synchronous response is received i.e., both parties occupy the same time and space, and the response is recorded by the implementer before the encounter is terminated. Any recording modality used by the programme implementer will be included, such as pen and paper or completion of an electronic form. For this review we will also include self-administered questionnaires as a subtype of in-person data collection, provided that; the data collection instrument is provided when the participant presents to a programme implementer in-person; the participant is asked to complete the data entry form; and the participant submits their responses before departing. Any non-hospital location will be accepted.

Voice call data collection includes real-time, telephone-based verbal exchanges between programme implementers and participants whereby SES data is elicited and recorded by the programme implementer using predefined questions. This category includes computer-assisted telephone interviews (CATI) – where the interviewer follows prompts on a computer screen – as well as non-computer assisted telephone interviews. Videocalls will be included as a subtype of voice-calls.

Automated telephone-based data collection includes any mobile-telephone-based asynchronous exchange of information whereby participants are sent a standardised text message (SMS), multimedia message (MMS), or automated phone call (sometimes called interactive voice response or 'IVR') and asked to provide SES data. Responses can be provided using the same modality or any other digital form e.g. entering details on a webpage. Interventions that require participants to engage with human programme implementers will be excluded. All forms of phrasing of the requests and responses will be included. We will exclude data collection approaches that require the download of third-party software, including email. For this review we will include web-surveys that can be accessed by a hyperlink, reasoning that all smartphones come with a pre-loaded browser.

Other terminology used in this review

'Community-based health programmes'. For the purpose of this review, health programmes are defined as organised activities to improve one or more health outcome(s) in a defined population. Community-based care encompasses all settings except hospitals. Other definitions of community-based care exclude primary care facilities,[12] but these will be included in this review, along with outreach/mobile clinics, community centres, schools, workplaces, and people's own homes.

For peer review only - http://bmjopen.bmj.com/site/about/guidelines.xhtml

'Programme implementers'. Anyone with a formal responsibility to collect data on behalf of the health programme will be dubbed a 'programme implementer' for the purpose of this review. This term will cover voluntary and paid staff, and all cadre types.

'Participants'. Any health programme beneficiary/recipient/client/patient that is asked to provide their SES data will be dubbed a 'participant' for the purpose of this review.

'Socioeconomic status' (SES). Socioeconomic status is a critically important but nebulous concept that pertains to social and economic standing within society.[13] It determines exposure to the social determinants of health; "the conditions in which people are born, grow, live, work, and age",[14] and relates to issues of privilege, power and control.[15] Almost all health outcomes are patterned according to SES, with the most disadvantaged populations experiencing the worst health outcomes.[16 17 18] SES is commonly measured using income, education, occupation, and other metrics such as wealth, caste, and place of residence. We will include all of these domains, as well as any other proxies that are identified by researchers as capturing SES.

'Low- and middle-income countries' (LMICs): Just as health inequalities exist within countries - driven by differential access to resources, power, privilege and control - the same set of factors drive international health inequalities. In 1975 Samuel Preston found that national life expectancy was tightly correlated with gross domestic product (GDP) by purchasing power parity, following a logarithmic path whereby small rises in GDP are initially associated with large gains in life expectancy, followed by increasingly diminishing returns.[19 20] In 1978 the World Bank first divided countries into 'low' and 'middle-income' groupings, based on gross national income (GNI) per capita. Whereas GDP captures the total value produced in a nation, GNI also includes net income received from overseas. Despite the fact that national finances are a fairly crude proxy,[21 22] many development agencies have come to use the World Bank categorisations to define eligibility for support. This review will use the World Bank analytic classifications for fiscal year 2021; defining LMICs as countries with gross national income (GNI) per capita ≤ $12,535[23] using the Atlas method.[24]

Objectives

We aim to systematically review the findings of empirical studies that have compared at least two different modalities for gathering SES data for community-based health programmes in terms of their resource requirements, performance characteristics, and acceptability to participants and service providers. Our findings should help programme managers make evidence-informed decisions when selecting the most appropriate modality for SES data collection.

Methods and analyses

This protocol is reported according to the relevant sections of the Preferred Reporting Items for Systematic Review and Meta-Analysis Protocols (PRISMA-P) guidelines.[25] It has been registered with PROSPERO, CRD42021251959.

Population

For this methodological paper, the 'population' is composed of studies rather than people, namely those that seek to compare two or more modalities for socioeconomic data collection from

For peer review only - http://bmjopen.bmj.com/site/about/guidelines.xhtml

individuals enrolled in health programmes. Studies that only report on only one mode of data collection will be excluded. Studies conducted in hospital-based ambulatory care facilities will be excluded.

Interventions

The interventions being studied are three different modalities for collecting socioeconomic data. The focus is on the modality of data collection (e.g. in-person vs voice call vs automated) rather than the content of the wording that is used to elicit information.

Three different modalities for SES data collection constitute the interventions of interests for this review: in-person, voice-call, and automated telephone systems, as defined in Box 1. We will exclude approaches that use a blend of modes to elicit SES data. We will also exclude studies where the SES questions and wording are not kept constant across modes .g. if a study asks about education via phone and face-to-face the question must be worded in the same way. This ensures that differences in response rates and other outcomes are only due to differences in mode of elicitation.

Studies that gather SES data at the household or community level will only be included if these data are used to make assumptions about the SES of identifiable individual participants enrolled (or due to be enrolled) in the service delivery programme of interest. Any two or more modalities can be studied. There is no index/gold-standard data collection modality. Interventions that bundle requests for SES data with requests for other data (e.g., broader demographic data) will be included, as long as separate results are reported for the SES data collection element. Interventions that use a blend of two or more modalities to request or receive data will be excluded. Studies that use email for data collection will be excluded.

Comparator

In-person, voice call, and automated telephone-based system attributes will be compared against each other. We will not include studies that only report outcomes for one modality i.e., comparisons are not possible. For each mode we will code the sub-type of data collection e.g. distinguishing between CATI and non-computer assisted telephone interviews. There is a risk that response rates will be influenced by other items in the survey, setting, and population. As such, our analysis will focus on outcome ratios between modes that pose the same questions in the same populations - rather than absolute levels as these may not be generalisable. We will report the wider context for each included study, and flag studies where SES questions are embedded within broader surveys that focus on taboo areas e.g. sexual behaviours or drug and alcohol use.

We will present outcomes for individual SES questions. We will only present data on identical questions asked using different modes i.e. if the wording is non-identical we will exclude the comparison from our analysis.

Primary outcomes

There are two groups of primary outcomes; performance characteristics and resource requirements. We will report these at the level of individual SES items.

Performance characteristics

For peer review only

- **Response rate**: number of completed SES items divided by the total number of elicitation attempts.

- **Completeness**: proportion of missing items. Completeness will be reported for individual SES questions, rather than for the whole questionnaire.

- **Equivalence:** agreement between the responses obtained from two or more different modalities. Recognising that equivalence can vary by question, we will report equivalence for each individual SES item. We will report equivalence figures if they aggregate multiple SES questions in a secondary analysis, however we will not report aggregate equivalence figures that mix SES items with non-SES items."
- Following Belisario and Gwaltney,[26] we will use comparisons of mean scores between modalities and/or correlations and/or measures of agreement - which include intra-class correlation (ICC) coefficients, Pearson product-moment correlations, Spearman rho and weighted Kappa coefficients.

Resource requirements

- **Time:** the time taken to gather SES data using each approach (range and mean).
- **Costs:** any financial data on the costs of operating the data collection approach will be included. Fixed costs include the costs of equipment, software, insurance, and personnel required to set up a given data elicitation modality. We will also include any ongoing support costs. We will aim to calculate the fixed and per-person costs to purchasers.

Secondary outcome

Acceptability to participants and service providers: Survey or interview results reporting on how programme implementers and participants feel about the data collection modality in terms of intrusiveness, ease of use, time requirement, and general acceptability, as well as perceived advantages, barriers, disadvantages, and additional costs presented by the beneficiaries, data collectors, or study authors. This includes an assessment of socioeconomic barriers to accessing the modalities.

Study types to be included

All empirical study designs that compare two or more data collection modalities will be included, for instance in-person vs SMS. Studies must compare modalities that have been used to gather data from participants. Studies that use simulated data, or data obtained from populations other than the intended beneficiaries will be excluded. Both quantitative and qualitative study designs will be included as long as they report on one or more of the outcomes of interest. Review articles will not be included, but their primary studies will be screened for potential inclusion.

Search methods for identification of studies

Search strategy

The search strategy will be built around three blocks: the three data collection modalities, SES concepts, and study design or study setting terms. The search will be limited to human studies

For peer review only

published since 1999: the year that it first became possible to send cross-network SMS messages. We will search for full-text studies published in any language. We will not include reports of studies published as conference abstracts. The full search strategies used for each database are presented in the Appendix. The search will be performed on 29th June 2021. We plan to complete the review by October 2022.

Electronic databases

We will search the following information resources: the Cochrane Library, MEDLINE, Embase and Global Health. We will search ClinicalTrials.gov and the WHO International Clinical Trials Registry Platform (ICTRP) for current and ongoing trials. OpenGrey will be searched for grey literature. The first 20 pages of Google Scholar will also be screened. We will check the reference lists of included studies and relevant systematic reviews to identify any additional potentially relevant reports of studies. Key authors will be contacted to uncover additional or upcoming studies.

Measures of effect

We will calculate mean differences for methodological performance between the modalities, as well as for time and cost differences. For equivalence we will follow Belisario[27] and Gwaltney,[28] using comparisons of mean scores between modalities and/or correlations and/or measures of agreement - which include intra-class correlation (ICC) coefficients, Pearson product-moment correlations, Spearman rho and weighted Kappa coefficients.

Data collection and analysis

Selection of studies

Initial screening of studies will be based on the information contained in their titles and abstracts, using online software (Covidence systematic review software, Veritas Health Innovation, Melbourne, Australia. Available at www.covidence.org). Studies that clearly do not meet the inclusion criteria will be excluded. The first 10% of papers will be screened by two reviewers collaboratively to align interpretation of the inclusion criteria and clarify the wording as appropriate. Any changes or amendments will be recorded. All remaining records will be screened independently by two reviewers. They will meet after every 10% batch of papers has been screened to discuss any issues. Any disagreements will be resolved through consensus-based discussion, or if necessary, discussion with a third reviewer.

We will obtain full texts for the potentially relevant papers. Two review authors will independently assess the papers against the inclusion criteria to determine their eligibility for inclusion. Non-English language papers will be translated into English. The review authors will resolve disagreements through consensus-based discussion, or if necessary, discussion with a third reviewer. The reviewers will record reasons for exclusion at the full-text screening stage. A PRISMA flow diagram will be completed to summarise the study selection process.[29]

Data extraction and management

Two review authors will independently extract study characteristics and data from the included studies using a custom Excel data extraction form based on the Cochrane template for RCTs and non RCTs.[30] The data extraction form will be piloted on 30 studies by two review authors and required

For peer review only - http://bmjopen.bmj.com/site/about/guidelines.xhtml

amendments will be made by consensus. We anticipate a broad scope of included studies, so data charting will be an iterative process throughout the review, with agreement calculated and discussed at regular intervals (after each 10% batch of studies) and the data extraction form will be amended as required. Any discrepancies will be resolved by discussion, and a third reviewer will be consulted if necessary.

The following data will be extracted:

- Article title
- Journal title
- Authors
- Country
- Language
- Publication year
- Type of study
- Focus of the service delivery programme
- Sociodemographic characteristics for the population served: age, sex, urban/rural, ethnicity, marital status
- Number of participants
- Questions used to assess SES
- Number of times SES data are collected from each participant
- Types of intervention, including:
    - modality
    - who gathers the SES data
    - when in the patient journey/programme
    - equipment used
    - who provides the data
    - synchronous or asynchronous
- Are continuous improvement methods are used to refine the data collection approach, based on performance data
- Types of comparison
- Types of outcome measures
- Outcomes: response rate, completeness, equivalence, time, and costs - as described above. We will extract all qualitative text provided on acceptability.

Risk of bias assessment for included studies

We will use the Cochrane RoB2 tool for randomised studies[31 32] and RoB-I for non-randomised studies.[33] Two reviewers will independently assess risk of bias. The review authors will resolve disagreements through a consensus-based decision, or if necessary, discussion with a third reviewer.

The risk of bias for each outcome across individual studies will be summarised as a narrative statement and supported by a risk of bias table. A review-level narrative summary of the risk of bias will also be provided.

Contacting study authors

For peer review only - http://bmjopen.bmj.com/site/about/guidelines.xhtml

We will contact study authors to request additional information and primary data where any aspect precludes the assessment of eligibility or inclusion in the data synthesis.

Strategy for data synthesis

If data are available, we will pool effect estimates using a random-effects model.[34]. However, we anticipate heterogeneity in study design, interventions and outcomes and therefore plan to use a narrative synthesis without meta-analysis approach, following the SWiM reporting guidelines from Campbell and colleagues.[35] We will stratify the synthesis by intervention type and outcome.

Assessment of heterogeneity

We will assess heterogeneity by considering study design, interventions, and outcomes.

Analysis of subgroups or subsets

We will assess whether response rates for each modality vary according to age, sex, urban/rural, ethnicity, and marital status where baseline data on the distribution of these characteristics within the general population are available.

We will perform a secondary analysis examining whether findings differ between high-income and low- and middle-income countries.

We will perform a secondary analysis where all studies found to be at high risk of bias will be excluded from the data synthesis.

Meta-biases

It is unlikely that we will be able to assess publication bias because it would require meta-analyses of ten or more studies, but if we do have such an analysis we will create a funnel plot.[36] Selective outcome reporting will be assessed by comparing protocols (where available) with published reports.

Assessment of certainty of evidence

Where possible, the GRADE criteria will be used to assess the certainty of the primary outcomes.[37 38] One review author will collate the evidence for each primary outcome and suggest initial ratings. These will be deliberated by a team of review authors who will reach a joint decision for each outcome. For RCTs evidence will be assumed to be high certainty and then will be downgraded due to risk of bias, inconsistency of results, indirectness of evidence, imprecision, publication bias. For observational studies, evidence starts at low-certainty but can be upgraded if there is a large effect, dose-response, gradient, or plausible confounding that decreases the magnitude of effect.

Patient and public involvement

No patient involved.

For peer review only - http://bmjopen.bmj.com/site/about/guidelines.xhtml

Ethics and dissemination

Ethical approval is not required, as our review will only include published and publicly accessible data.

We will publish our findings in an open-access, peer-reviewed journal and develop an accessible summary of the results for website posting and stakeholder meetings. Data generated from this review will be made available upon reasonable request.

Authors' contributions

LA planned the study with SM, IG, DB, APM, MJ, DM, MK, JR and AB. IG and LA designed the search terms. IG conducted the search. LA and SM conducted screening, extraction and quality scoring. LA wrote the first draft. All authors contributed to analysing and interpreting the findings. All authors read and approved the final protocol.

Funding

This work was supported by the Wellcome Trust and NIHR grant number 215633/Z/19/Z. The study was sponsored by the London School of Hygiene & Tropical Medicine.

The funders had no role in the development of the protocol and will not play any role in the execution of the systematic review.

Competing interests

None declared.

Patient consent for publication

Not required.

For peer review only

References

[1] Marmot M. Social determinants of health inequalities. The Lancet. 2005 Mar 19;365(9464):1099-104.

[2] Hart JT. The inverse care law. The Lancet. 1971 Feb 27;297(7696):405-12.

[3] WHO. Constitution of the World Health Organization. 1948. Available at: https://apps.who.int/gb/bd/PDF/bd47/EN/constitution-en.pdf?ua=1 [Accessed 11/05/2021]

[4] United Nations General Assembly. The Sustainable Development Goals. Available at: https://www.who.int/topics/sustainable-development-goals/targets/en/ [Accessed 11/05/2021]

[5] Ng M, Fullman N, Dieleman JL, Flaxman AD, Murray CJ, Lim SS. Effective coverage: a metric for monitoring universal health coverage. PLoS Med. 2014 Sep 22;11(9):e1001730.

[6] Rodney AM, Hill PS. Achieving equity within universal health coverage: a narrative review of progress and resources for measuring success. International journal for equity in health. 2014 Dec;13(1):1-8.

[7] Guo S, Carvajal-Aguirre L, Victora CG, Barros AJ, Wehrmeister FC, Vidaletti LP, Gupta G, Matin MZ, Rutter P. Equitable coverage? The roles of the private and public sectors in providing maternal, newborn and child health interventions in South Asia. BMJ global health. 2019 Aug 1;4(4):e001495.

[8] Carey G, Crammond B, De Leeuw E. Towards health equity: a framework for the application of proportionate universalism. International journal for equity in health. 2015 Dec;14(1):1-8.

[9] Burton MJ, Ramke J, Marques AP, Bourne RR, Congdon N, Jones I, Tong BA, Arunga S, Bachani D, Bascaran C, Bastawrous A. The Lancet global health Commission on global eye health: vision beyond 2020. The Lancet Global Health. 2021 Apr 1;9(4):e489-551.

[10] Allen LN, Smith RW, Simmons-Jones F, Roberts N, Honney R, Currie J. Addressing social determinants of noncommunicable diseases in primary care: a systematic review. Bulletin of the World Health Organization. 2020 Nov 1;98(11):754.

[11] Peek Vision. Available at: https://www.peekvision.org/ [Accessed 14/05/2021].

[12] The King's Fund. Community health services explained. Available at: https://www.kingsfund.org.uk/publications/community-health-services-explained [Accessed 13/05/2021].

[13] American Psychological Association. Socioeconomic status. Available at: https://www.apa.org/topics/socioeconomic-status [Accessed 13/05/2021].

[14] WHO. Closing the gap in a generation: The commission on the social determinants of health. Available at: https://www.who.int/publications/i/item/WHO-IER-CSDH-08.1 [Accessed 13/05/2021].

[15] World Health Organization. Social determinants of health. Available at: https://www.who.int/news-room/q-a-detail/social-determinants-of-health-key-concepts [Accessed 13/05/2021].

For peer review only - http://bmjopen.bmj.com/site/about/guidelines.xhtml

[16] World Health Organization. Social determinants of health: the solid facts. World Health Organization. Regional Office for Europe; 2003.

[17] Marmot M. Social determinants of health inequalities. The Lancet. 2005 Mar 19;365(9464):1099-104.

[18] American Psychological Association. Socioeconomic status. Available at: https://www.apa.org/topics/socioeconomic-status [Accessed 13/05/2021].

[19] Preston SH. The changing relation between mortality and level of economic development. Population studies. 1975 Jul 1;29(2):231-48.

[20] Preston SH. The changing relation between mortality and level of economic development.

Int J Epidemiol. 2007; 36: 484-490

[21] Bloom DE, Canning D. Commentary: The Preston Curve 30 years on: still sparking fires. Int J Epidemiol. 2007 Jun 1;36(3):498-9.

[22] World Bank. Why use GNI per capita to classify economies into income groupings? Available at: https://datahelpdesk.worldbank.org/knowledgebase/articles/378831-why-use-gni-per-capita-to-classify-economies-into [Accessed 24/06/2021].

[23] World Bank. Country and lending groups: fiscal year 2021. Available at: https://datahelpdesk.worldbank.org/knowledgebase/articles/906519-world-bank-country-and-lending-groups [Accessed 13/05/2021].

[24] World Bank. The Altas method - detailed methodology. Available at: https://datahelpdesk.worldbank.org/knowledgebase/articles/378832-what-is-the-world-bank-atlas-method [Accessed 13/05/2021].

[25] Shamseer L, Moher D, Clarke M, Ghersi D, Liberati A, Petticrew M, Shekelle P, Stewart L, PRISMA-P Group. Preferred reporting items for systematic review and meta-analysis protocols (PRISMA-P) 2015: elaboration and explanation. BMJ. 2015 Jan 2;349(jan02 1):g7647.

[26] Belisario JS, Jamsek J, Huckvale K, O'Donoghue J, Morrison CP, Car J. Comparison of self-administered survey questionnaire responses collected using mobile apps versus other methods. Cochrane database of systematic reviews. 2015(7).

[27] Belisario JS, Jamsek J, Huckvale K, O'Donoghue J, Morrison CP, Car J. Comparison of self-administered survey questionnaire responses collected using mobile apps versus other methods. Cochrane database of systematic reviews. 2015(7).

[28] Gwaltney CJ, Shields AL, Shiffman S. Equivalence of electronic and paper-and-pencil administration of patient-reported outcome measures: a meta-analytic review. Value in Health 2008;11(2):322-33.

[29] Page et al. The PRISMA 2020 statement: an updated guideline for reporting systematic reviews. BMJ 2021; 372.

For peer review only

[30] Cochrane. Template collection form for RCTs and non RCTs. Available at: https://training.cochrane.org/sites/training.cochrane.org/files/public/uploads/resources/downloadable_resources/English/Collecting%20data%20-%20form%20for%20RCTs%20and%20non-RCTs.doc

[31] Risk of Bias tool. Available at: https://www.riskofbias.info/. [Accessed 18/05/2021].

[32] Sterne JAC, Savović J, Page MJ, Elbers RG, Blencowe NS, Boutron I, Cates CJ, Cheng H-Y, Corbett MS, Eldridge SM, Hernán MA, Hopewell S, Hróbjartsson A, Junqueira DR, Jüni P, Kirkham JJ, Lasserson T, Li T, McAleenan A, Reeves BC, Shepperd S, Shrier I, Stewart LA, Tilling K, White IR, Whiting PF, Higgins JPT. RoB 2: a revised tool for assessing risk of bias in randomised trials. BMJ 2019; 366: l4898.

[33]Sterne JAC, Hernán MA, Reeves BC, Savović J, Berkman ND, Viswanathan M, Henry D, Altman DG, Ansari MT, Boutron I, Carpenter JR, Chan AW, Churchill R, Deeks JJ, Hróbjartsson A, Kirkham J, Jüni P, Loke YK, Pigott TD, Ramsay CR, Regidor D, Rothstein HR, Sandhu L, Santaguida PL, Schünemann HJ, Shea B, Shrier I, Tugwell P, Turner L, Valentine JC, Waddington H, Waters E, Wells GA, Whiting PF, Higgins JPT. ROBINS-I: a tool for assessing risk of bias in non-randomized studies of interventions. BMJ 2016; 355; i4919; doi: 10.1136/bmj.i4919.

[34] Deeks JJ, Higgins JPT, Altman DG. Cochrane Handbook Chapter 10: Analysing data and undertaking meta-analyses. Available at: https://training.cochrane.org/handbook/current/chapter-10 [Accessed 18/05/2021].

[35] Campbell M, McKenzie JE, Sowden A, Katikireddi SV, Brennan SE, Ellis S, Hartmann-Boyce J, Ryan R, Shepperd S, Thomas J, Welch V, Thomson H. Synthesis without meta-analysis (SWiM) in systematic reviews: reporting guideline BMJ 2020;368:l6890 http://dx.doi.org/10.1136/bmj.l6890

[36] Page MJ, Higgins JPT, Sterne JAC. Cochrane Handbook Chapter 13: Assessing risk of bias due to missing results in a synthesis. Available at: https://training.cochrane.org/handbook/current/chapter-13 [Accessed 18/05/2021].

[37] Murad MH, Mustafa RA, Schünemann HJ, Sultan S, Santesso N. Rating the certainty in evidence in the absence of a single estimate of effect. BMJ Evidence-Based Medicine. 2017 Jun 1;22(3):85-7.

[38] GRADE. The GRADE handbook. Available at: https://gdt.gradepro.org/app/handbook/handbook.html [Accessed 18/05/2021].

For peer review only

Supplementary File

Search strategies

MEDLINE

Ovid MEDLINE(R) and Epub Ahead of Print, In-Process, In-Data-Review & Other Non-Indexed Citations and Daily

1. Telephone/
2. (telephone$ or phone$).tw.
3. ((voice or phone) adj1 call$).tw.
4. (phone adj2 interview$).tw.
5. Cell Phones/
6. Smartphone/
7. (phone$ adj1 (smart or cell)).tw.
8. (smartphone$ or cellphone$).tw.
9. (mobile adj2 (phone$ or device$)).tw.
10. or/1-9
11. Text Messaging/
12. (text or texts or texting).tw.
13. MMS.tw.
14. SMS.tw.
15. short message service.tw.
16. multimedia message service.tw.
17. (automated adj2 (telephone$ or text$ or message$ or questionnaire$)).tw.
18. (telephone adj1 administered adj1 questionnaire$).tw.
19. or/11-18
20. Interviews as Topic/
21. Patient Health Questionnaire/
22. Self Report/
23. (in adj1 person$).tw.
24. (in adj1 person$ adj4 (interview$ or survey$ or question$)).tw.
25. (face adj2 face adj4 (interview$ or survey$ or question$)).tw.
26. (face-to-face adj4 (interview$ or survey$ or question$)).tw.
27. or/20-26
28. 10 and 19
29. 10 and 27
30. 19 and 27
31. 10 and 19 and 27
32. 28 or 29 or 30 or 31
33. Vulnerable populations/ or socioeconomic factors/ or poverty/ or social class/ or Healthcare Disparities/ or Health Status Disparities/ or Poverty areas/ or Urban population/
34. (equit$ or inequit$ or inequalit$ or disparit$ or equality).tw.
35. (ethnic$ or race or racial$ or caste$).tw.

For peer review only

36. ((social$ or socio-economic or socioeconomic or economic or structural or material) adj3 (advantage$ or disadvantage$ or exclude$ or exclusion or include$ or inclusion or status or position or gradient$ or hierarch$ or class$ or determinant$)).tw.
37. (health adj3 (gap$ or gradient$ or hierarch$)).tw.
38. exp education/ or educational status/ or employment/ or income/ or occupations/ or social conditions/
39. (SES or SEP or sociodemographic$ or socio-demographic$ or demographic$ or income or wealth$ or poverty or affluen$).tw.
40. (educat$ adj3 (level$ or attain$ or status or well or better)).tw.
41. (occupation or unemploy$).tw.
42. (home owner$ or tenure).tw.
43. (household adj2 (income or wealth or status)).tw.
44. ((well or better or worse) adj2 off).tw.
45. or/33-43
46. Community Health Planning/
47. Community Health Services/
48. Community Health Nursing/
49. National Health Programs/
50. State Medicine/
51. Regional Health Planning/
52. Health Planning/
53. Health Plan Implementation/
54. Health Planning Guidelines/
55. Health Care Reform/
56. Health Resources/
57. Health Priorities/
58. Health Services Research/
59. "health services needs and demand"/
60. Needs Assessment/
61. State Health Plans/
62. Regional Health Planning/
63. Primary Health Care/
64. Health Services, Indigenous/
65. Rural Health Services/
66. Mobile Health Units/
67. randomized controlled trial/ or controlled clinical trials as topic/ or randomized controlled trials as topic/
68. (randomized or randomised or randomly or RCT).tw.
69. outcome assessment, health care/
70. comparative study/ or evaluation studies/ or meta-analysis/ or multicenter study/ or "systematic review"/ or validation studies/
71. epidemiologic studies/ or follow-up studies/ or longitudinal studies/ or prospective studies/ or controlled before-after studies/
72. or/46-71
73. 32 and 45 and 72

For peer review only

74. limit 73 to yr="1999 -Current"

Cochrane Library

#1 MeSH descriptor: [Telephone] this term only
#2 telephone* or phone*
#3 (voice or phone) near/1 call*
#4 phone near/2 interview*
#5 MeSH descriptor: [Cell Phone] this term only
#6 MeSH descriptor: [Smartphone] this term only
#7 phone* near/1 (smart or cell)
#8 smartphone* or cellphone*
#9 mobile near/2 (phone* or device*)
#10 #1 or #2 or #3 or #4 or #5 or #6 or #7 or #8 or #9
#11 MeSH descriptor: [Text Messaging] this term only
#12 text or texts or texting
#13 MMS or SMS
#14 "multimedia message service"
#15 "short message service"
#16 automated near/2 (telephone* or text* or message* or questionnaire*)
#17 telephone near/1 administered near/1 questionnaire*
#18 #11 or #12 or #13 or #14 or #15 or #16 or #17
#19 MeSH descriptor: [Interviews as Topic] this term only
#20 MeSH descriptor: [Patient Health Questionnaire] this term only
#21 MeSH descriptor: [Self Report] this term only
#22 in near/1 person*
#23 (in near/1 person* near/4 (interview* or survey* or question*))
#24 (face near/2 face near/4 (interview* or survey* or question*))
#25 (face-to-face near/4 (interview* or survey* or question*))
#26 #19 or #20 or #21 or #22 or #23 or #24 or #25
#27 #10 and #18
#28 #10 and #26
#29 #18 and #26
#30 #10 and #18 and #26
#31 #27 or #28 or #29 or #30
#32 MeSH descriptor: [Socioeconomic Factors] this term only
#33 MeSH descriptor: [Poverty] this term only
#34 MeSH descriptor: [Social Class] this term only
#35 MeSH descriptor: [Vulnerable Populations] this term only
#36 MeSH descriptor: [Healthcare Disparities] this term only
#37 MeSH descriptor: [Health Status Disparities] this term only
#38 MeSH descriptor: [Poverty Areas] this term only
#39 MeSH descriptor: [Urban Population] this term only
#40 equit* or inequit* or inequalit* or disparit* or equality

For peer review only

#41 ethnic* or race or racial* or caste*
#42 (social* or socio-economic or socioeconomic or economic or structural or material) near/3 (advantage* or disadvantage* or exclude* or exclusion or include* or inclusion or status or position or gradient* or hierarch* or class* or determinant*)
#43 health near/3 (gap* or gradient* or hierarch*)
#44 MeSH descriptor: [Education] explode all trees
#45 MeSH descriptor: [Educational Status] this term only
#46 MeSH descriptor: [Employment] this term only
#47 MeSH descriptor: [Income] this term only
#48 MeSH descriptor: [Occupations] this term only
#49 MeSH descriptor: [Social Conditions] this term only
#50 SES or SEP or sociodemographic* or socio-demographic* or income or wealth* or poverty or affluen*
#51 educat* near/3 (level* or attain* or status or well or better)
#52 occupation or unemploy*
#53 home owner* or tenure
#54 household near/2 (income or wealth or status)
#55 (well or better or worse) near/2 off
#56 #32 or #33 or #34 or #35 or #36 or #37 or #38 or #39 or #40 or #41 or #42 or #43 or #44 or #45 or #46 or #47 or #48 or #49 or #50 or #51 or #52 or #53 or #54 or #55
#57 MeSH descriptor: [Community Health Planning] this term only
#58 MeSH descriptor: [Community Health Services] this term only
#59 MeSH descriptor: [Community Health Nursing] explode all trees
#60 MeSH descriptor: [National Health Programs] this term only
#61 MeSH descriptor: [State Medicine] explode all trees
#62 MeSH descriptor: [Regional Health Planning] this term only
#63 MeSH descriptor: [Health Planning] this term only
#64 MeSH descriptor: [Health Plan Implementation] this term only
#65 MeSH descriptor: [Health Planning Guidelines] this term only
#66 MeSH descriptor: [Health Care Reform] this term only
#67 MeSH descriptor: [Health Resources] this term only
#68 MeSH descriptor: [Health Priorities] this term only
#69 MeSH descriptor: [Health Services Research] this term only
#70 MeSH descriptor: [Health Services Needs and Demand] this term only
#71 MeSH descriptor: [Needs Assessment] this term only
#72 MeSH descriptor: [State Health Plans] this term only
#73 MeSH descriptor: [Regional Health Planning] this term only
#74 MeSH descriptor: [Primary Health Care] this term only
#75 MeSH descriptor: [Health Services, Indigenous] this term only
#76 MeSH descriptor: [Rural Health Services] this term only
#77 MeSH descriptor: [Mobile Health Units] this term only
#78 #57 or #58 or #59 or #60 or #61 or #62 or #63 or #64 or #65 or #66 or #67 or #68 or #69 or #70 or #71 or #72 or #73 or #74 or #75 or #76 or #77
#79 #31 and #56 and #78 with Publication Year from 1999 to 2021, in Trials

For peer review only

Embase

1. telephone/
2. telephone interview/
3. (telephone$ or phone$).tw.
4. ((voice or phone) adj1 call$).tw.
5. (phone adj2 interview$).tw.
6. mobile phone/
7. smartphone/
8. (phone$ adj1 (smart or cell)).tw.
9. (smartphone$ or cellphone$).tw.
10. (mobile adj2 (phone$ or device$)).tw.
11. or/1-10
12. text messaging/
13. (text or texts or texting).tw.
14. MMS.tw.
15. SMS.tw.
16. multimedia message service.tw.
17. short message service.tw.
18. (automated adj2 (telephone$ or text$ or message$ or questionnaire$)).tw.
19. (telephone adj1 administered adj1 questionnaire$).tw.
20. or/12-19
21. interview/
22. (in adj1 person$).tw.
23. (in adj1 person$ adj4 (interview$ or survey$ or question$)).tw.
24. (face adj2 face adj4 (interview$ or survey$ or question$)).tw.
25. (face-to-face adj4 (interview$ or survey$ or question$)).tw.
26. or/21-25
27. 11 and 20
28. 11 and 26
29. 20 and 26
30. 11 and 20 and 26
31. 27 or 28 or 29 or 30
32. socioeconomics/
33. poverty/
34. social status/
35. social class/
36. vulnerable population/
37. health care disparity/
38. health disparity/
39. urban population/
40. (equit$ or inequit$ or inequalit$ or disparit$ or equality).tw.
41. (ethnic$ or race or racial$ or caste$).tw.

For peer review only

42. ((social$ or socio-economic or socioeconomic or economic or structural or material) adj3 (advantage$ or disadvantage$ or exclude$ or exclusion or include$ or inclusion or status or position or gradient$ or hierarch$ or class$ or determinant$)).tw.
43. (health adj3 (gap$ or gradient$ or hierarch$)).tw.
44. education/
45. educational status/
46. employment/
47. employment status/
48. unemployment/
49. household income/ or family income/ or income/
50. occupation/
51. (SES or SEP or sociodemographic$ or socio-demographic$ or demographic$ or income or wealth$ or poverty or affluen$).tw.
52. (educat$ adj3 (level$ or attain$ or status or well or better)).tw.
53. (occupation or unemploy$).tw.
54. (home owner$ or tenure).tw.
55. (household adj2 (income or wealth or status)).tw.
56. ((well or better or worse) adj2 off).tw.
57. or/32-56
58. public health/
59. health care planning/
60. community care/
61. community health nursing/
62. national health service/
63. health care policy/
64. health services research/
65. health service/
66. primary health care/
67. indigenous health care/
68. rural health care/
69. randomized controlled trial/ or controlled clinical trial/ or "randomized controlled trial (topic)"/
70. (randomized or randomised or randomly or RCT).tw.
71. outcome assessment/
72. comparative study/
73. evaluation study/
74. "systematic review"/ or "systematic review (topic)"/ or meta analysis/
75. epidemiology/
76. prospective study/
77. longitudinal study/
78. follow up/
79. or/58-78
80. 31 and 57 and 79
81. limit 80 to yr="1999 -Current"

For peer review only

Global Health

1. mobile telephones/ or telephones/
2. (telephone$ or phone$).tw.
3. ((voice or phone) adj1 call$).tw.
4. (phone adj2 interview$).tw.
5. (phone$ adj1 (smart or cell)).tw.
6. (smartphone$ or cellphone$).tw.
7. (mobile adj2 (phone$ or device$)).tw.
8. or/1-7
9. (text or texts or texting).tw.
10. (MMS or SMS).tw.
11. multimedia message service.tw.
12. short message service.tw.
13. (automated adj2 (telephone$ or text$ or message$ or questionnaire$)).tw.
14. (telephone adj1 administered adj1 questionnaire$).tw.
15. or/9-14
16. interviews/
17. (in adj1 person).tw.
18. (in adj1 person adj4 (interview$ or survey$ or question$)).tw.
19. (face adj2 face adj4 (interview$ or survey$ or question$)).tw.
20. (face-to-face adj4 (interview$ or survey$ or question$)).tw.
21. or/16-20
22. 7 and 15
23. 7 and 21
24. 15 and 21
25. 7 and 15 and 21
26. 22 or 23 or 24 or 25
27. socioeconomic status/ or socioeconomics/
28. poverty/
29. exp social classes/ or caste/ or social inequalities/ or social mobility/
30. urban population/
31. (equit$ or inequit$ or inequalit$ or disparit$ or equality).tw.
32. (ethnic$ or race or racial$ or caste$).tw.
33. ((social$ or socio-economic or socioeconomic or economic or structural or material) adj3 (advantage$ or disadvantage$ or exclude$ or exclusion or include$ or inclusion or status or position or gradient$ or hierarch$ or class$ or determinant$)).tw.
34. (health adj3 (gap$ or gradient$ or hierarch$)).tw.
35. education/
36. employment/
37. occupations/
38. income/ or household income/
39. living conditions/
40. (SES or SEP or sociodemographic$ or socio-demographic$ or demographic$ or income or wealth$ or poverty or affluen$).tw.

For peer review only

41. (educat$ adj3 (level$ or attain$ or status or well or better)).tw.
42. (occupation or unemploy$).tw.
43. (home owner$ or tenure).tw.
44. (household adj2 (income or wealth or status)).tw.
45. ((well or better or worse) adj2 off).tw.
46. or/27-45
47. 26 and 46
48. limit 47 to yr="1999 -Current"

ClinicalTrials.gov

Search 1
socioeconomic AND (telephone OR phone) AND (interview OR face-to-face OR in-person) AND community | Interventional Studies

Search 2
socioeconomic AND (telephone OR phone) AND (text OR SMS OR MMS) AND community | Interventional Studies

Search 3
socioeconomic AND (text OR SMS OR MMS) AND (interview OR face-to-face OR in-person) AND community | Interventional Studies

WHO ICTRP

Search 1
socioeconomic AND telephone AND interview AND community

Search 2
socioeconomic AND telephone AND text AND community

Search 3
socioeconomic AND text AND interview AND community

OpenGrey

socioeconomic AND (telephone OR phone OR text OR interview OR face-to-face OR in-person) AND community

For peer review only

BMJ Open

Performance and resource requirements of in-person vs voice call vs automated telephone-based socioeconomic data collection modalities for community-based health programmes: a systematic review protocol

Journal:	*BMJ Open*
Manuscript ID	bmjopen-2021-057410.R2
Article Type:	Protocol
Date Submitted by the Author:	21-Mar-2022
Complete List of Authors:	Allen, Luke; London School of Hygiene & Tropical Medicine, Department of Clinical Research<br>Mackinnon, Shona; University of Glasgow<br>Gordon, Iris; London School of Hygiene & Tropical Medicine, Department of Clinical Research<br>Blane, David; University of Glasgow<br>Marques, Ana Patricia; London School of Hygiene and Tropical Medicine International Centre for Eye Health,<br>Gichuhi, Stephen; University of Nairobi<br>Mwangi, Alice; Operation Eyesight<br>Burton, Matthew J; London School of Hygiene & Tropical Medicine, Department of Clinical Research<br>Bolster, Nigel; Peek Vision<br>Macleod, David; London School of Hygiene & Tropical Medicine<br>Kim, Min; London School of Hygiene and Tropical Medicine Faculty of Infectious and Tropical Diseases<br>Ramke, Jacqueline; London School of Hygiene & Tropical Medicine, Department of Clinical Research<br>Bastawrous, Andrew; London School of Hygiene & Tropical Medicine
**Primary Subject Heading**:	Public health
Secondary Subject Heading:	Health services research
Keywords:	PRIMARY CARE, PUBLIC HEALTH, Epidemiology < TROPICAL MEDICINE

SCHOLARONE™
Manuscripts

Performance and resource requirements of in-person vs voice call vs automated telephone-based socioeconomic data collection modalities for community-based health programmes: a systematic review protocol

Authors
Dr Luke N Allen (corresponding author)
International Centre for Eye Health, Department of Clinical Research, London School of Hygiene & Tropical Medicine, Keppel St, London WC1E 7HT
drlukeallen@gmail.com
ORCID ID: 0000-0003-2750-3575

Dr Shona Mackinnon
NHS Education for Scotland
shonamackinnon@doctors.org.uk

Ms Iris Gordon
International Centre for Eye Health, Department of Clinical Research, London School of Hygiene & Tropical Medicine, Keppel St, London WC1E 7HT
ORCID: 0001-8143-8132
iris.gordon@lshtm.ac.uk

Dr David Blane
Institute of Health and Wellbeing, University of Glasgow
David.Blane@glasgow.ac.uk
ORCID ID: 0000-0002-3872-3621

Dr Ana Patricia Marques
International Centre for Eye Health, Department of Clinical Research, London School of Hygiene & Tropical Medicine, Keppel St, London WC1E 7HT
Patricia.Marques@lshtm.ac.uk
ORCID ID: 0000-0001-8242-7021

Dr Stephen Gichuhi
MBChB, M.Med (Ophth), MBA, MSc (Epid), PhD, FCOphth(ECSA)
Senior Lecturer & Consultant Ophthalmologist, Chairman, Department of Ophthalmology, University of Nairobi
stephen.gichuhi@lshtm.ac.uk

Ms Alice Mwangi
Operation Eyesight
mwangia@operationeyesight.com

For peer review only

Prof Matthew J. Burton
International Centre for Eye Health, Department of Clinical Research, London School of Hygiene & Tropical Medicine, Keppel St, London WC1E 7HT
matthew.burton@lshtm.ac.uk

Dr Nigel Bolster
International Centre for Eye Health, Department of Clinical Research, London School of Hygiene & Tropical Medicine, Keppel St, London WC1E 7HT

Peek Vision, 90a High Street, Berkhamsted, Hertfordshire, England HP4 2BL
nigel@peekvision.org
ORCID: 0000-0001-6607-1723

Dr David Macleod
International Statistics & Epidemiology Group, Department of Infectious Disease Epidemiology, London School of Hygiene & Tropical Medicine, Keppel St, London WC1E 7HT
david.macleod@lshtm.ac.uk

Ms Min Kim
International Centre for Eye Health, Department of Clinical Research, London School of Hygiene & Tropical Medicine, Keppel St, London WC1E 7HT
min.kim@lshtm.ac.uk

Assoc Prof Jacqueline Ramke
International Centre for Eye Health, Department of Clinical Research, London School of Hygiene & Tropical Medicine, Keppel St, London WC1E 7HT
School of Optometry and Vision Science, University of Auckland, Auckland, New Zealand
Jacqueline.Ramke@lshtm.ac.uk

Assoc Prof Andrew Bastawrous
International Centre for Eye Health, Department of Clinical Research, London School of Hygiene & Tropical Medicine, Keppel St, London WC1E 7HT
Andrew.bastawrous@lshtm.ac.uk

For peer review only

Abstract

Introduction

Gathering data on socioeconomic status (SES) is a prerequisite for any health programme that aims to assess and improve the equitable distribution of its outcomes. Many different modalities can be used to collect SES data, ranging from (1) face-to-face elicitation, to (2) telephone-administered questionnaires, to (3) automated text message-based systems. The relative costs and perceived benefits to patients and providers of these different data collection approaches is unknown. This protocol is for a systematic review that aims to compare the resource requirements, performance characteristics, and acceptability to participants and service providers of these three approaches to collect SES data from those enrolled in health programmes.

Methods and analysis

An information specialist will conduct searches on the Cochrane Library, MEDLINE, Embase, Global Health, Clinicaltrials.gov, the WHO ICTRP and OpenGrey. All databases will be searched from 1999 to present with no language limits used. We will also search Google Scholar and check the reference lists of relevant articles for further potentially eligible studies. Any empirical study design will be eligible if it compares two or more modalities to elicit SES data from the following three; in-person, voice call, or automated phone-based systems. Two reviewers will independently screen titles, abstracts and full-text articles; and complete data extraction. For each study we will extract data on the modality characteristics, primary outcomes (response rate and equivalence) and secondary outcomes (time, costs, and acceptability to patients and providers). We will synthesise findings thematically without meta-analysis.

Ethics and dissemination

Ethical approval is not required, as our review will include published and publicly accessible data. This review is part of a project to improve equitable access to eye care services in low- and middle-income countries. However, the findings will be useful to policymakers and programme managers in a range of health- and non-health settings. We will publish our findings in a peer-reviewed journal and develop an accessible summary of results for website posting and stakeholder meetings.

Registration

This review is registered with PROSPERO CRD42021251959

Strengths and limitations of this study

- As far as we are aware, this review will be the first to directly compare three commonly used data collection modalities for the collection of SES data.
- The review will be comprehensive, covering published and grey literature in any language.
- This review will be robust, using independent dual review at every stage, and following best-practice guidelines.
- There may only be a small number of articles in the literature that compare the different modalities head-to-head and provide data on the outcomes of interest.

For peer review only

Introduction

Rationale

Inequalities in health are pervasive and stubbornly persistent. Individuals with lower levels of income, education, and social status tend to experience the worst health outcomes irrespective of where they are in the world.[1] In 1971 Julian Tudor Hart observed that the availability of good medical care tends to vary inversely with the need for it in the population served.[2] This inverse care law manifests in the majority of global health and development programmes where individuals with the lowest socioeconomic status (SES) tend to face the highest barriers in accessing care and are the least likely to attain good outcomes.

Recognising marked inter- and intra-national disparities in health outcomes, the World Health Organisation (WHO) was constituted in 1948 with the mandate of advancing ‘health for all’.[3] The contemporary manifestation of this mission is encapsulated in the concept of Universal Health Coverage (and Sustainable Development Goal target 3.8[4]) which seeks to extend coverage to disenfranchised groups. Emerging emphases on attaining *effective* coverage,[5] and *equitable* coverage[6 7] seek to shift the success criteria from supply-side provision of services to demand-side receipt of effective services according to need. These trends are underpinned by the principle of ‘proportionate universalism’: seeking to improve the health of all, with the greatest gains experienced by those with the greatest needs.[8] There is also an increasing interest in understanding the distribution of programme benefits across socio-demographic groups - for instance women, those living in rural locations, and those living in conditions of poverty.[9]

All attempts to boost equity in service provision are predicated on adequate collection and analysis of sociodemographic data. Previous work has demonstrated that sociodemographic data can be collected using a variety of modalities in the community setting including in-person, telephone voice calls, and using automated telephone-based systems[10] (Box 1). However, as far as we are aware, the relative costs and benefits of the different modalities have not been studied, including the skills, equipment, time and financial resources required and acceptability to data collectors and service beneficiaries.

This review aims to answer the research question ‘how do three common SES data collection modalities compare in terms of performance characteristics, resource requirements and acceptability to participants and service providers?’ Selecting an appropriate and cost-effective modality is an important first step towards advancing equitable effective service coverage.

The findings of this review will directly inform the development of school and community-based eye health screening programmes that operates in several low- and middle-income countries (LMICs) including Botswana, Ethiopia, Kenya, Nepal, Pakistan, Tanzania, Uganda and Zimbabwe.[11] However, the collection of SES data is relevant for a much wider range of global health programmes, as well as non-health programmes aimed at improving educational, agricultural, gender equity, and economic outcomes, among others.

Descriptions of the interventions

For peer review only - http://bmjopen.bmj.com/site/about/guidelines.xhtml

Three different modalities for SES data collection constitute the interventions of interest for this review: in-person; voice call; and automated telephone data collection. Box 1 provides the definition for each.

Box 1: Definitions of the three data collection approaches used in this review

In-person data collection includes any form of exchange between a programme implementer and a participant or their responsible guardian, whereby the programme implementer asks predefined questions to ascertain the participants' socioeconomic status and a synchronous response is received i.e., both parties occupy the same time and space, and the response is recorded by the implementer before the encounter is terminated. Any recording modality used by the programme implementer will be included, such as pen and paper or completion of an electronic form. For this review we will also include self-administered questionnaires as a subtype of in-person data collection, provided that; the data collection instrument is provided when the participant presents to a programme implementer in-person; the participant is asked to complete the data entry form; and the participant submits their responses before departing. Any non-hospital location will be accepted.

Voice call data collection includes real-time, telephone-based verbal exchanges between programme implementers and participants whereby SES data is elicited and recorded by the programme implementer using predefined questions. This category includes computer-assisted telephone interviews (CATI) – where the interviewer follows prompts on a computer screen – as well as non-computer assisted telephone interviews. Videocalls will be included as a subtype of voice-calls.

Automated telephone-based data collection includes any mobile-telephone-based asynchronous exchange of information whereby participants are sent a standardised text message (SMS), multimedia message (MMS), or automated phone call (sometimes called interactive voice response or 'IVR') and asked to provide SES data. Responses can be provided using the same modality or any other digital form e.g. entering details on a webpage. Interventions that require participants to engage with human programme implementers will be excluded. All forms of phrasing of the requests and responses will be included. We will exclude data collection approaches that require the download of third-party software, including email. For this review we will include web-surveys that can be accessed by a hyperlink, reasoning that all smartphones come with a pre-loaded browser.

Other terminology used in this review

'Community-based health programmes'. For the purpose of this review, health programmes are defined as organised activities to improve one or more health outcome(s) in a defined population. Community-based care encompasses all settings except hospitals. Other definitions of community-based care exclude primary care facilities,[12] but these will be included in this review, along with outreach/mobile clinics, community centres, schools, workplaces, and people's own homes.

For peer review only - http://bmjopen.bmj.com/site/about/guidelines.xhtml

‘Programme implementers’. Anyone with a formal responsibility to collect data on behalf of the health programme will be dubbed a ‘programme implementer’ for the purpose of this review. This term will cover voluntary and paid staff, and all cadre types.

‘Participants’. Any health programme beneficiary/recipient/client/patient that is asked to provide their SES data will be dubbed a ‘participant’ for the purpose of this review.

‘Socioeconomic status’ (SES). Socioeconomic status is a critically important but nebulous concept that pertains to social and economic standing within society.[13] It determines exposure to the social determinants of health; “the conditions in which people are born, grow, live, work, and age”,[14] and relates to issues of privilege, power and control.[15] Almost all health outcomes are patterned according to SES, with the most disadvantaged populations experiencing the worst health outcomes.[16 17 18] SES is commonly measured using income, education, occupation, and other metrics such as wealth, caste, and place of residence. We will include all of these domains, as well as any other proxies that are identified by researchers as capturing SES.

‘Low- and middle-income countries’ (LMICs): Just as health inequalities exist within countries - driven by differential access to resources, power, privilege and control - the same set of factors drive international health inequalities. In 1975 Samuel Preston found that national life expectancy was tightly correlated with gross domestic product (GDP) by purchasing power parity, following a logarithmic path whereby small rises in GDP are initially associated with large gains in life expectancy, followed by increasingly diminishing returns.[19 20] In 1978 the World Bank first divided countries into ‘low’ and ‘middle-income’ groupings, based on gross national income (GNI) per capita. Whereas GDP captures the total value produced in a nation, GNI also includes net income received from overseas. Despite the fact that national finances are a fairly crude proxy,[21 22] many development agencies have come to use the World Bank categorisations to define eligibility for support. This review will use the World Bank analytic classifications for fiscal year 2021; defining LMICs as countries with gross national income (GNI) per capita ≤ $12,535[23] using the Atlas method.[24]

Objectives

We aim to systematically review the findings of empirical studies that have compared at least two different modalities for gathering SES data for community-based health programmes in terms of their resource requirements, performance characteristics, and acceptability to participants and service providers. Our findings should help programme managers make evidence-informed decisions when selecting the most appropriate modality for SES data collection.

Methods and analyses

This protocol is reported according to the relevant sections of the Preferred Reporting Items for Systematic Review and Meta-Analysis Protocols (PRISMA-P) guidelines.[25] It has been registered with PROSPERO, CRD42021251959.

Population

For this methodological paper, the ‘population’ is composed of studies rather than people, namely those that seek to compare two or more modalities for socioeconomic data collection from

For peer review only - http://bmjopen.bmj.com/site/about/guidelines.xhtml

individuals enrolled in health programmes. Studies that only report on only one mode of data collection will be excluded. Studies conducted in hospital-based ambulatory care facilities will be excluded.

Interventions

The interventions being studied are three different modalities for collecting socioeconomic data. The focus is on the modality of data collection (e.g. in-person vs voice call vs automated) rather than the content of the wording that is used to elicit information.

Three different modalities for SES data collection constitute the interventions of interests for this review: in-person, voice-call, and automated telephone systems, as defined in Box 1. We will exclude approaches that use a blend of modes to elicit SES data. We will also exclude studies where the SES questions and wording are not kept constant across modes .g. if a study asks about education via phone and face-to-face the question must be worded in the same way. This ensures that differences in response rates and other outcomes are only due to differences in mode of elicitation.

Studies that gather SES data at the household or community level will only be included if these data are used to make assumptions about the SES of identifiable individual participants enrolled (or due to be enrolled) in the service delivery programme of interest. Any two or more modalities can be studied. There is no index/gold-standard data collection modality. Interventions that bundle requests for SES data with requests for other data (e.g., broader demographic data) will be included, as long as separate results are reported for the SES data collection element. Interventions that use a blend of two or more modalities to request or receive data will be excluded. Studies that use email for data collection will be excluded.

Comparator

In-person, voice call, and automated telephone-based system attributes will be compared against each other. We will not include studies that only report outcomes for one modality i.e., comparisons are not possible. For each mode we will code the sub-type of data collection e.g. distinguishing between CATI and non-computer assisted telephone interviews. There is a risk that response rates will be influenced by other items in the survey, setting, and population. As such, our analysis will focus on outcome ratios between modes that pose the same questions in the same populations - rather than absolute levels as these may not be generalisable. We will report the wider context for each included study, and flag studies where SES questions are embedded within broader surveys that focus on taboo areas e.g. sexual behaviours or drug and alcohol use.

We will present outcomes for individual SES questions. We will only present data on identical questions asked using different modes i.e. if the wording is non-identical we will exclude the comparison from our analysis.

Primary outcomes

There are two groups of primary outcomes; performance characteristics and resource requirements. We will report these at the level of individual SES items.

Performance characteristics

For peer review only - http://bmjopen.bmj.com/site/about/guidelines.xhtml

- **Response rate**: number of completed SES items divided by the total number of elicitation attempts. This will be calculated at the level of each individual SES item.

- **Equivalence:** agreement between the responses obtained from two or more different modalities. Recognising that equivalence can vary by question, we will report equivalence for each individual SES item. We will report equivalence figures if they aggregate multiple SES questions in a secondary analysis, however we will not report aggregate equivalence figures that mix SES items with non-SES items."
- Following Belisario and Gwaltney,[26] we will use comparisons of mean scores between modalities and/or correlations and/or measures of agreement - which include intra-class correlation (ICC) coefficients, Pearson product-moment correlations, Spearman rho and weighted Kappa coefficients.

Resource requirements

- **Time:** the time taken to gather SES data using each approach (range and mean).
- **Costs:** any financial data on the costs of operating the data collection approach will be included. Fixed costs include the costs of equipment, software, insurance, and personnel required to set up a given data elicitation modality. We will also include any ongoing support costs. We will aim to calculate the fixed and per-person costs to purchasers.

Secondary outcome

Acceptability to participants and service providers: Survey or interview results reporting on how programme implementers and participants feel about the data collection modality in terms of intrusiveness, ease of use, time requirement, and general acceptability, as well as perceived advantages, barriers, disadvantages, and additional costs presented by the beneficiaries, data collectors, or study authors. This includes an assessment of socioeconomic barriers to accessing the modalities.

Study types to be included

All empirical study designs that compare two or more data collection modalities will be included, for instance in-person vs SMS. Studies must compare modalities that have been used to gather data from participants. Studies that use simulated data, or data obtained from populations other than the intended beneficiaries will be excluded. Both quantitative and qualitative study designs will be included as long as they report on one or more of the outcomes of interest. Review articles will not be included, but their primary studies will be screened for potential inclusion.

Search methods for identification of studies

Search strategy

The search strategy will be built around three blocks: the three data collection modalities, SES concepts, and study design or study setting terms. The search will be limited to human studies published since 1999: the year that it first became possible to send cross-network SMS messages. We will search for full-text studies published in any language. We will not include reports of studies

For peer review only - http://bmjopen.bmj.com/site/about/guidelines.xhtml

published as conference abstracts. The full search strategies used for each database are presented in the Appendix. The search will be performed on 29th June 2021. We plan to complete the review by October 2022.

Electronic databases

We will search the following information resources: the Cochrane Library, MEDLINE, Embase and Global Health. We will search ClinicalTrials.gov and the WHO International Clinical Trials Registry Platform (ICTRP) for current and ongoing trials. OpenGrey will be searched for grey literature. The first 20 pages of Google Scholar will also be screened. We will check the reference lists of included studies and relevant systematic reviews to identify any additional potentially relevant reports of studies. Key authors will be contacted to uncover additional or upcoming studies.

Measures of effect

We will calculate mean differences for methodological performance between the modalities, as well as for time and cost differences. For equivalence we will follow Belisario[27] and Gwaltney,[28] using comparisons of mean scores between modalities and/or correlations and/or measures of agreement - which include intra-class correlation (ICC) coefficients, Pearson product-moment correlations, Spearman rho and weighted Kappa coefficients.

Data collection and analysis

Selection of studies

Initial screening of studies will be based on the information contained in their titles and abstracts, using online software (Covidence systematic review software, Veritas Health Innovation, Melbourne, Australia. Available at www.covidence.org). Studies that clearly do not meet the inclusion criteria will be excluded. The first 10% of papers will be screened by two reviewers collaboratively to align interpretation of the inclusion criteria and clarify the wording as appropriate. Any changes or amendments will be recorded. All remaining records will be screened independently by two reviewers. They will meet after every 10% batch of papers has been screened to discuss any issues. Any disagreements will be resolved through consensus-based discussion, or if necessary, discussion with a third reviewer.

We will obtain full texts for the potentially relevant papers. Two review authors will independently assess the papers against the inclusion criteria to determine their eligibility for inclusion. Non-English language papers will be translated into English. The review authors will resolve disagreements through consensus-based discussion, or if necessary, discussion with a third reviewer. The reviewers will record reasons for exclusion at the full-text screening stage. A PRISMA flow diagram will be completed to summarise the study selection process.[29]

Data extraction and management

Two review authors will independently extract study characteristics and data from the included studies using a custom Excel data extraction form based on the Cochrane template for RCTs and non RCTs.[30] The data extraction form will be piloted on 30 studies by two review authors and required amendments will be made by consensus. We anticipate a broad scope of included studies, so data charting will be an iterative process throughout the review, with agreement calculated and discussed

For peer review only

at regular intervals (after each 10% batch of studies) and the data extraction form will be amended as required. Any discrepancies will be resolved by discussion, and a third reviewer will be consulted if necessary.

The following data will be extracted:

- Article title
- Journal title
- Authors
- Country
- Language
- Publication year
- Type of study
- Focus of the service delivery programme
- Sociodemographic characteristics for the population served: age, sex, urban/rural, ethnicity, marital status
- Number of participants
- Questions used to assess SES
- Number of times SES data are collected from each participant
- Types of intervention, including:
  - modality
  - who gathers the SES data
  - when in the patient journey/programme
  - equipment used
  - who provides the data
  - synchronous or asynchronous
- Are continuous improvement methods are used to refine the data collection approach, based on performance data
- Types of comparison
- Types of outcome measures
- Outcomes: response rate, completeness, equivalence, time, and costs - as described above. We will extract all qualitative text provided on acceptability.

Risk of bias assessment for included studies

We will use the Cochrane RoB2 tool for randomised studies[31 32] and RoB-I for non-randomised studies.[33] Two reviewers will independently assess risk of bias. The review authors will resolve disagreements through a consensus-based decision, or if necessary, discussion with a third reviewer.

The risk of bias for each outcome across individual studies will be summarised as a narrative statement and supported by a risk of bias table. A review-level narrative summary of the risk of bias will also be provided.

Contacting study authors

We will contact study authors to request additional information and primary data where any aspect precludes the assessment of eligibility or inclusion in the data synthesis.

For peer review only

Strategy for data synthesis

If data are available, we will pool effect estimates using a random-effects model.[34]. However, we anticipate heterogeneity in study design, interventions and outcomes and therefore plan to use a narrative synthesis without meta-analysis approach, following the SWiM reporting guidelines from Campbell and colleagues.[35] We will stratify the synthesis by intervention type and outcome. Studies found to be at high risk of bias will be excluded from the synthesis.

Assessment of heterogeneity

We will assess heterogeneity by considering study design, interventions, and outcomes.

Analysis of subgroups or subsets

We will assess whether response rates for each modality vary according to age, sex, urban/rural, ethnicity, and marital status where baseline data on the distribution of these characteristics within the general population are available.

We will perform a secondary analysis examining whether findings differ between high-income and low- and middle-income countries.

We will perform a secondary analysis that includes all studies found to be at high risk of bias inincluded the data synthesis.

Meta-biases

It is unlikely that we will be able to assess publication bias because it would require meta-analyses of ten or more studies, but if we do have such an analysis we will create a funnel plot.[36] Selective outcome reporting will be assessed by comparing protocols (where available) with published reports.

Assessment of certainty of evidence

Where possible, the GRADE criteria will be used to assess the certainty of the primary outcomes.[37 38] One review author will collate the evidence for each primary outcome and suggest initial ratings. These will be deliberated by a team of review authors who will reach a joint decision for each outcome. For RCTs evidence will be assumed to be high certainty and then will be downgraded due to risk of bias, inconsistency of results, indirectness of evidence, imprecision, publication bias. For observational studies, evidence starts at low-certainty but can be upgraded if there is a large effect, dose-response, gradient, or plausible confounding that decreases the magnitude of effect.

Patient and public involvement

No patient involved.

Ethics and dissemination

Ethical approval is not required, as our review will only include published and publicly accessible data.

For peer review only - http://bmjopen.bmj.com/site/about/guidelines.xhtml

We will publish our findings in an open-access, peer-reviewed journal and develop an accessible summary of the results for website posting and stakeholder meetings. Data generated from this review will be made available upon reasonable request.

Authors' contributions

LA conceptualised and planned the study with SM, IG, DB, APM, MJ, DM, MK, JR and AB. IG and LA designed the search terms. IG conducted the search. LA and SM conducted screening, extraction and quality scoring. DB APM SG AM MB NB DM MK JR AB helped to analyse and interpret the initial findings. LA wrote the first draft with SM. IG DB APM SG AM MB NB DM MK JR AB critically revised iterations of the manuscript. All authors read and approved the final protocol.

Funding

This work was supported by the Wellcome Trust and NIHR grant number 215633/Z/19/Z. The study was sponsored by the London School of Hygiene & Tropical Medicine.

The funders had no role in the development of the protocol and will not play any role in the execution of the systematic review.

Competing interests

None declared.

Patient consent for publication

Not required.

For peer review only

References

[1] Marmot M. Social determinants of health inequalities. The Lancet. 2005 Mar 19;365(9464):1099-104.

[2] Hart JT. The inverse care law. The Lancet. 1971 Feb 27;297(7696):405-12.

[3] WHO. Constitution of the World Health Organization. 1948. Available at: https://apps.who.int/gb/bd/PDF/bd47/EN/constitution-en.pdf?ua=1 [Accessed 11/05/2021]

[4] United Nations General Assembly. The Sustainable Development Goals. Available at: https://www.who.int/topics/sustainable-development-goals/targets/en/ [Accessed 11/05/2021]

[5] Ng M, Fullman N, Dieleman JL, Flaxman AD, Murray CJ, Lim SS. Effective coverage: a metric for monitoring universal health coverage. PLoS Med. 2014 Sep 22;11(9):e1001730.

[6] Rodney AM, Hill PS. Achieving equity within universal health coverage: a narrative review of progress and resources for measuring success. International journal for equity in health. 2014 Dec;13(1):1-8.

[7] Guo S, Carvajal-Aguirre L, Victora CG, Barros AJ, Wehrmeister FC, Vidaletti LP, Gupta G, Matin MZ, Rutter P. Equitable coverage? The roles of the private and public sectors in providing maternal, newborn and child health interventions in South Asia. BMJ global health. 2019 Aug 1;4(4):e001495.

[8] Carey G, Crammond B, De Leeuw E. Towards health equity: a framework for the application of proportionate universalism. International journal for equity in health. 2015 Dec;14(1):1-8.

[9] Burton MJ, Ramke J, Marques AP, Bourne RR, Congdon N, Jones I, Tong BA, Arunga S, Bachani D, Bascaran C, Bastawrous A. The Lancet global health Commission on global eye health: vision beyond 2020. The Lancet Global Health. 2021 Apr 1;9(4):e489-551.

[10] Allen LN, Smith RW, Simmons-Jones F, Roberts N, Honney R, Currie J. Addressing social determinants of noncommunicable diseases in primary care: a systematic review. Bulletin of the World Health Organization. 2020 Nov 1;98(11):754.

[11] Peek Vision. Available at: https://www.peekvision.org/ [Accessed 14/05/2021].

[12] The King's Fund. Community health services explained. Available at: https://www.kingsfund.org.uk/publications/community-health-services-explained [Accessed 13/05/2021].

[13] American Psychological Association. Socioeconomic status. Available at: https://www.apa.org/topics/socioeconomic-status [Accessed 13/05/2021].

[14] WHO. Closing the gap in a generation: The commission on the social determinants of health. Available at: https://www.who.int/publications/i/item/WHO-IER-CSDH-08.1 [Accessed 13/05/2021].

[15] World Health Organization. Social determinants of health. Available at: https://www.who.int/news-room/q-a-detail/social-determinants-of-health-key-concepts [Accessed 13/05/2021].

For peer review only - http://bmjopen.bmj.com/site/about/guidelines.xhtml

[16] World Health Organization. Social determinants of health: the solid facts. World Health Organization. Regional Office for Europe; 2003.

[17] Marmot M. Social determinants of health inequalities. The Lancet. 2005 Mar 19;365(9464):1099-104.

[18] American Psychological Association. Socioeconomic status. Available at: https://www.apa.org/topics/socioeconomic-status [Accessed 13/05/2021].

[19] Preston SH. The changing relation between mortality and level of economic development. Population studies. 1975 Jul 1;29(2):231-48.

[20] Preston SH. The changing relation between mortality and level of economic development.

Int J Epidemiol. 2007; 36: 484-490

[21] Bloom DE, Canning D. Commentary: The Preston Curve 30 years on: still sparking fires. Int J Epidemiol. 2007 Jun 1;36(3):498-9.

[22] World Bank. Why use GNI per capita to classify economies into income groupings? Available at: https://datahelpdesk.worldbank.org/knowledgebase/articles/378831-why-use-gni-per-capita-to-classify-economies-into [Accessed 24/06/2021].

[23] World Bank. Country and lending groups: fiscal year 2021. Available at: https://datahelpdesk.worldbank.org/knowledgebase/articles/906519-world-bank-country-and-lending-groups [Accessed 13/05/2021].

[24] World Bank. The Altas method - detailed methodology. Available at: https://datahelpdesk.worldbank.org/knowledgebase/articles/378832-what-is-the-world-bank-atlas-method [Accessed 13/05/2021].

[25] Shamseer L, Moher D, Clarke M, Ghersi D, Liberati A, Petticrew M, Shekelle P, Stewart L, PRISMA-P Group. Preferred reporting items for systematic review and meta-analysis protocols (PRISMA-P) 2015: elaboration and explanation. BMJ. 2015 Jan 2;349(jan02 1):g7647.

[26] Belisario JS, Jamsek J, Huckvale K, O'Donoghue J, Morrison CP, Car J. Comparison of self-administered survey questionnaire responses collected using mobile apps versus other methods. Cochrane database of systematic reviews. 2015(7).

[27] Belisario JS, Jamsek J, Huckvale K, O'Donoghue J, Morrison CP, Car J. Comparison of self-administered survey questionnaire responses collected using mobile apps versus other methods. Cochrane database of systematic reviews. 2015(7).

[28] Gwaltney CJ, Shields AL, Shiffman S. Equivalence of electronic and paper-and-pencil administration of patient-reported outcome measures: a meta-analytic review. Value in Health 2008;11(2):322-33.

[29] Page et al. The PRISMA 2020 statement: an updated guideline for reporting systematic reviews. BMJ 2021; 372.

For peer review only

[30] Cochrane. Template collection form for RCTs and non RCTs. Available at: https://training.cochrane.org/sites/training.cochrane.org/files/public/uploads/resources/downloadable_resources/English/Collecting%20data%20-%20form%20for%20RCTs%20and%20non-RCTs.doc

[31] Risk of Bias tool. Available at: https://www.riskofbias.info/. [Accessed 18/05/2021].

[32] Sterne JAC, Savović J, Page MJ, Elbers RG, Blencowe NS, Boutron I, Cates CJ, Cheng H-Y, Corbett MS, Eldridge SM, Hernán MA, Hopewell S, Hróbjartsson A, Junqueira DR, Jüni P, Kirkham JJ, Lasserson T, Li T, McAleenan A, Reeves BC, Shepperd S, Shrier I, Stewart LA, Tilling K, White IR, Whiting PF, Higgins JPT. RoB 2: a revised tool for assessing risk of bias in randomised trials. BMJ 2019; 366: l4898.

[33]Sterne JAC, Hernán MA, Reeves BC, Savović J, Berkman ND, Viswanathan M, Henry D, Altman DG, Ansari MT, Boutron I, Carpenter JR, Chan AW, Churchill R, Deeks JJ, Hróbjartsson A, Kirkham J, Jüni P, Loke YK, Pigott TD, Ramsay CR, Regidor D, Rothstein HR, Sandhu L, Santaguida PL, Schünemann HJ, Shea B, Shrier I, Tugwell P, Turner L, Valentine JC, Waddington H, Waters E, Wells GA, Whiting PF, Higgins JPT. ROBINS-I: a tool for assessing risk of bias in non-randomized studies of interventions. BMJ 2016; 355; i4919; doi: 10.1136/bmj.i4919.

[34] Deeks JJ, Higgins JPT, Altman DG. Cochrane Handbook Chapter 10: Analysing data and undertaking meta-analyses. Available at: https://training.cochrane.org/handbook/current/chapter-10 [Accessed 18/05/2021].

[35] Campbell M, McKenzie JE, Sowden A, Katikireddi SV, Brennan SE, Ellis S, Hartmann-Boyce J, Ryan R, Shepperd S, Thomas J, Welch V, Thomson H. Synthesis without meta-analysis (SWiM) in systematic reviews: reporting guideline BMJ 2020;368:l6890 http://dx.doi.org/10.1136/bmj.l6890

[36] Page MJ, Higgins JPT, Sterne JAC. Cochrane Handbook Chapter 13: Assessing risk of bias due to missing results in a synthesis. Available at: https://training.cochrane.org/handbook/current/chapter-13 [Accessed 18/05/2021].

[37] Murad MH, Mustafa RA, Schünemann HJ, Sultan S, Santesso N. Rating the certainty in evidence in the absence of a single estimate of effect. BMJ Evidence-Based Medicine. 2017 Jun 1;22(3):85-7.

[38] GRADE. The GRADE handbook. Available at: https://gdt.gradepro.org/app/handbook/handbook.html [Accessed 18/05/2021].

For peer review only

Supplementary File

Search strategies

MEDLINE

Ovid MEDLINE(R) and Epub Ahead of Print, In-Process, In-Data-Review & Other Non-Indexed Citations and Daily

1. Telephone/
2. (telephone$ or phone$).tw.
3. ((voice or phone) adj1 call$).tw.
4. (phone adj2 interview$).tw.
5. Cell Phones/
6. Smartphone/
7. (phone$ adj1 (smart or cell)).tw.
8. (smartphone$ or cellphone$).tw.
9. (mobile adj2 (phone$ or device$)).tw.
10. or/1-9
11. Text Messaging/
12. (text or texts or texting).tw.
13. MMS.tw.
14. SMS.tw.
15. short message service.tw.
16. multimedia message service.tw.
17. (automated adj2 (telephone$ or text$ or message$ or questionnaire$)).tw.
18. (telephone adj1 administered adj1 questionnaire$).tw.
19. or/11-18
20. Interviews as Topic/
21. Patient Health Questionnaire/
22. Self Report/
23. (in adj1 person$).tw.
24. (in adj1 person$ adj4 (interview$ or survey$ or question$)).tw.
25. (face adj2 face adj4 (interview$ or survey$ or question$)).tw.
26. (face-to-face adj4 (interview$ or survey$ or question$)).tw.
27. or/20-26
28. 10 and 19
29. 10 and 27
30. 19 and 27
31. 10 and 19 and 27
32. 28 or 29 or 30 or 31
33. Vulnerable populations/ or socioeconomic factors/ or poverty/ or social class/ or Healthcare Disparities/ or Health Status Disparities/ or Poverty areas/ or Urban population/
34. (equit$ or inequit$ or inequalit$ or disparit$ or equality).tw.
35. (ethnic$ or race or racial$ or caste$).tw.

For peer review only

36. ((social$ or socio-economic or socioeconomic or economic or structural or material) adj3 (advantage$ or disadvantage$ or exclude$ or exclusion or include$ or inclusion or status or position or gradient$ or hierarch$ or class$ or determinant$)).tw.
37. (health adj3 (gap$ or gradient$ or hierarch$)).tw.
38. exp education/ or educational status/ or employment/ or income/ or occupations/ or social conditions/
39. (SES or SEP or sociodemographic$ or socio-demographic$ or demographic$ or income or wealth$ or poverty or affluen$).tw.
40. (educat$ adj3 (level$ or attain$ or status or well or better)).tw.
41. (occupation or unemploy$).tw.
42. (home owner$ or tenure).tw.
43. (household adj2 (income or wealth or status)).tw.
44. ((well or better or worse) adj2 off).tw.
45. or/33-43
46. Community Health Planning/
47. Community Health Services/
48. Community Health Nursing/
49. National Health Programs/
50. State Medicine/
51. Regional Health Planning/
52. Health Planning/
53. Health Plan Implementation/
54. Health Planning Guidelines/
55. Health Care Reform/
56. Health Resources/
57. Health Priorities/
58. Health Services Research/
59. "health services needs and demand"/
60. Needs Assessment/
61. State Health Plans/
62. Regional Health Planning/
63. Primary Health Care/
64. Health Services, Indigenous/
65. Rural Health Services/
66. Mobile Health Units/
67. randomized controlled trial/ or controlled clinical trials as topic/ or randomized controlled trials as topic/
68. (randomized or randomised or randomly or RCT).tw.
69. outcome assessment, health care/
70. comparative study/ or evaluation studies/ or meta-analysis/ or multicenter study/ or "systematic review"/ or validation studies/
71. epidemiologic studies/ or follow-up studies/ or longitudinal studies/ or prospective studies/ or controlled before-after studies/
72. or/46-71
73. 32 and 45 and 72

For peer review only

74. limit 73 to yr="1999 -Current"

Cochrane Library

#1 MeSH descriptor: [Telephone] this term only
#2 telephone* or phone*
#3 (voice or phone) near/1 call*
#4 phone near/2 interview*
#5 MeSH descriptor: [Cell Phone] this term only
#6 MeSH descriptor: [Smartphone] this term only
#7 phone* near/1 (smart or cell)
#8 smartphone* or cellphone*
#9 mobile near/2 (phone* or device*)
#10 #1 or #2 or #3 or #4 or #5 or #6 or #7 or #8 or #9
#11 MeSH descriptor: [Text Messaging] this term only
#12 text or texts or texting
#13 MMS or SMS
#14 "multimedia message service"
#15 "short message service"
#16 automated near/2 (telephone* or text* or message* or questionnaire*)
#17 telephone near/1 administered near/1 questionnaire*
#18 #11 or #12 or #13 or #14 or #15 or #16 or #17
#19 MeSH descriptor: [Interviews as Topic] this term only
#20 MeSH descriptor: [Patient Health Questionnaire] this term only
#21 MeSH descriptor: [Self Report] this term only
#22 in near/1 person*
#23 (in near/1 person* near/4 (interview* or survey* or question*))
#24 (face near/2 face near/4 (interview* or survey* or question*))
#25 (face-to-face near/4 (interview* or survey* or question*))
#26 #19 or #20 or #21 or #22 or #23 or #24 or #25
#27 #10 and #18
#28 #10 and #26
#29 #18 and #26
#30 #10 and #18 and #26
#31 #27 or #28 or #29 or #30
#32 MeSH descriptor: [Socioeconomic Factors] this term only
#33 MeSH descriptor: [Poverty] this term only
#34 MeSH descriptor: [Social Class] this term only
#35 MeSH descriptor: [Vulnerable Populations] this term only
#36 MeSH descriptor: [Healthcare Disparities] this term only
#37 MeSH descriptor: [Health Status Disparities] this term only
#38 MeSH descriptor: [Poverty Areas] this term only
#39 MeSH descriptor: [Urban Population] this term only
#40 equit* or inequit* or inequalit* or disparit* or equality

For peer review only

#41 ethnic* or race or racial* or caste*
#42 (social* or socio-economic or socioeconomic or economic or structural or material) near/3 (advantage* or disadvantage* or exclude* or exclusion or include* or inclusion or status or position or gradient* or hierarch* or class* or determinant*)
#43 health near/3 (gap* or gradient* or hierarch*)
#44 MeSH descriptor: [Education] explode all trees
#45 MeSH descriptor: [Educational Status] this term only
#46 MeSH descriptor: [Employment] this term only
#47 MeSH descriptor: [Income] this term only
#48 MeSH descriptor: [Occupations] this term only
#49 MeSH descriptor: [Social Conditions] this term only
#50 SES or SEP or sociodemographic* or socio-demographic* or income or wealth* or poverty or affluen*
#51 educat* near/3 (level* or attain* or status or well or better)
#52 occupation or unemploy*
#53 home owner* or tenure
#54 household near/2 (income or wealth or status)
#55 (well or better or worse) near/2 off
#56 #32 or #33 or #34 or #35 or #36 or #37 or #38 or #39 or #40 or #41 or #42 or #43 or #44 or #45 or #46 or #47 or #48 or #49 or #50 or #51 or #52 or #53 or #54 or #55
#57 MeSH descriptor: [Community Health Planning] this term only
#58 MeSH descriptor: [Community Health Services] this term only
#59 MeSH descriptor: [Community Health Nursing] explode all trees
#60 MeSH descriptor: [National Health Programs] this term only
#61 MeSH descriptor: [State Medicine] explode all trees
#62 MeSH descriptor: [Regional Health Planning] this term only
#63 MeSH descriptor: [Health Planning] this term only
#64 MeSH descriptor: [Health Plan Implementation] this term only
#65 MeSH descriptor: [Health Planning Guidelines] this term only
#66 MeSH descriptor: [Health Care Reform] this term only
#67 MeSH descriptor: [Health Resources] this term only
#68 MeSH descriptor: [Health Priorities] this term only
#69 MeSH descriptor: [Health Services Research] this term only
#70 MeSH descriptor: [Health Services Needs and Demand] this term only
#71 MeSH descriptor: [Needs Assessment] this term only
#72 MeSH descriptor: [State Health Plans] this term only
#73 MeSH descriptor: [Regional Health Planning] this term only
#74 MeSH descriptor: [Primary Health Care] this term only
#75 MeSH descriptor: [Health Services, Indigenous] this term only
#76 MeSH descriptor: [Rural Health Services] this term only
#77 MeSH descriptor: [Mobile Health Units] this term only
#78 #57 or #58 or #59 or #60 or #61 or #62 or #63 or #64 or #65 or #66 or #67 or #68 or #69 or #70 or #71 or #72 or #73 or #74 or #75 or #76 or #77
#79 #31 and #56 and #78 with Publication Year from 1999 to 2021, in Trials

For peer review only

Embase

1. telephone/
2. telephone interview/
3. (telephone$ or phone$).tw.
4. ((voice or phone) adj1 call$).tw.
5. (phone adj2 interview$).tw.
6. mobile phone/
7. smartphone/
8. (phone$ adj1 (smart or cell)).tw.
9. (smartphone$ or cellphone$).tw.
10. (mobile adj2 (phone$ or device$)).tw.
11. or/1-10
12. text messaging/
13. (text or texts or texting).tw.
14. MMS.tw.
15. SMS.tw.
16. multimedia message service.tw.
17. short message service.tw.
18. (automated adj2 (telephone$ or text$ or message$ or questionnaire$)).tw.
19. (telephone adj1 administered adj1 questionnaire$).tw.
20. or/12-19
21. interview/
22. (in adj1 person$).tw.
23. (in adj1 person$ adj4 (interview$ or survey$ or question$)).tw.
24. (face adj2 face adj4 (interview$ or survey$ or question$)).tw.
25. (face-to-face adj4 (interview$ or survey$ or question$)).tw.
26. or/21-25
27. 11 and 20
28. 11 and 26
29. 20 and 26
30. 11 and 20 and 26
31. 27 or 28 or 29 or 30
32. socioeconomics/
33. poverty/
34. social status/
35. social class/
36. vulnerable population/
37. health care disparity/
38. health disparity/
39. urban population/
40. (equit$ or inequit$ or inequalit$ or disparit$ or equality).tw.
41. (ethnic$ or race or racial$ or caste$).tw.

For peer review only

42. ((social$ or socio-economic or socioeconomic or economic or structural or material) adj3 (advantage$ or disadvantage$ or exclude$ or exclusion or include$ or inclusion or status or position or gradient$ or hierarch$ or class$ or determinant$)).tw.
43. (health adj3 (gap$ or gradient$ or hierarch$)).tw.
44. education/
45. educational status/
46. employment/
47. employment status/
48. unemployment/
49. household income/ or family income/ or income/
50. occupation/
51. (SES or SEP or sociodemographic$ or socio-demographic$ or demographic$ or income or wealth$ or poverty or affluen$).tw.
52. (educat$ adj3 (level$ or attain$ or status or well or better)).tw.
53. (occupation or unemploy$).tw.
54. (home owner$ or tenure).tw.
55. (household adj2 (income or wealth or status)).tw.
56. ((well or better or worse) adj2 off).tw.
57. or/32-56
58. public health/
59. health care planning/
60. community care/
61. community health nursing/
62. national health service/
63. health care policy/
64. health services research/
65. health service/
66. primary health care/
67. indigenous health care/
68. rural health care/
69. randomized controlled trial/ or controlled clinical trial/ or "randomized controlled trial (topic)"/
70. (randomized or randomised or randomly or RCT).tw.
71. outcome assessment/
72. comparative study/
73. evaluation study/
74. "systematic review"/ or "systematic review (topic)"/ or meta analysis/
75. epidemiology/
76. prospective study/
77. longitudinal study/
78. follow up/
79. or/58-78
80. 31 and 57 and 79
81. limit 80 to yr="1999 -Current"

For peer review only

Global Health

1. mobile telephones/ or telephones/
2. (telephone$ or phone$).tw.
3. ((voice or phone) adj1 call$).tw.
4. (phone adj2 interview$).tw.
5. (phone$ adj1 (smart or cell)).tw.
6. (smartphone$ or cellphone$).tw.
7. (mobile adj2 (phone$ or device$)).tw.
8. or/1-7
9. (text or texts or texting).tw.
10. (MMS or SMS).tw.
11. multimedia message service.tw.
12. short message service.tw.
13. (automated adj2 (telephone$ or text$ or message$ or questionnaire$)).tw.
14. (telephone adj1 administered adj1 questionnaire$).tw.
15. or/9-14
16. interviews/
17. (in adj1 person).tw.
18. (in adj1 person adj4 (interview$ or survey$ or question$)).tw.
19. (face adj2 face adj4 (interview$ or survey$ or question$)).tw.
20. (face-to-face adj4 (interview$ or survey$ or question$)).tw.
21. or/16-20
22. 7 and 15
23. 7 and 21
24. 15 and 21
25. 7 and 15 and 21
26. 22 or 23 or 24 or 25
27. socioeconomic status/ or socioeconomics/
28. poverty/
29. exp social classes/ or caste/ or social inequalities/ or social mobility/
30. urban population/
31. (equit$ or inequit$ or inequalit$ or disparit$ or equality).tw.
32. (ethnic$ or race or racial$ or caste$).tw.
33. ((social$ or socio-economic or socioeconomic or economic or structural or material) adj3 (advantage$ or disadvantage$ or exclude$ or exclusion or include$ or inclusion or status or position or gradient$ or hierarch$ or class$ or determinant$)).tw.
34. (health adj3 (gap$ or gradient$ or hierarch$)).tw.
35. education/
36. employment/
37. occupations/
38. income/ or household income/
39. living conditions/
40. (SES or SEP or sociodemographic$ or socio-demographic$ or demographic$ or income or wealth$ or poverty or affluen$).tw.

For peer review only

41. (educat$ adj3 (level$ or attain$ or status or well or better)).tw.
42. (occupation or unemploy$).tw.
43. (home owner$ or tenure).tw.
44. (household adj2 (income or wealth or status)).tw.
45. ((well or better or worse) adj2 off).tw.
46. or/27-45
47. 26 and 46
48. limit 47 to yr="1999 -Current"

ClinicalTrials.gov

Search 1
socioeconomic AND (telephone OR phone) AND (interview OR face-to-face OR in-person) AND community | Interventional Studies

Search 2
socioeconomic AND (telephone OR phone) AND (text OR SMS OR MMS) AND community | Interventional Studies

Search 3
socioeconomic AND (text OR SMS OR MMS) AND (interview OR face-to-face OR in-person) AND community | Interventional Studies

WHO ICTRP

Search 1
socioeconomic AND telephone AND interview AND community

Search 2
socioeconomic AND telephone AND text AND community

Search 3
socioeconomic AND text AND interview AND community

OpenGrey

socioeconomic AND (telephone OR phone OR text OR interview OR face-to-face OR in-person) AND community

For peer review only
